# Multi-Transcriptome-Informed Network Pharmacology Reveals Novel Biomarkers and Therapeutic Candidates for Parkinson’s Disease

**DOI:** 10.3390/genes16121459

**Published:** 2025-12-07

**Authors:** Md. Al Amin Pappu, Md. Alamin, Md Al Noman, Most. Humaira Sultana, Md. Foysal Ahmed, Md. Sanoar Hossain, Md. Abdul Latif, Md. Fahim Faysal, AKM Azad, Salem A. Alyami, Naif Alotaibi, Md. Nurul Haque Mollah

**Affiliations:** 1 Bioinformatics Lab (Dry), Department of Statistics, University of Rajshahi, Rajshahi 6205, Bangladesh; 2 Department of Mathematics and Physics, School of Engineering & Physical Sciences, North South University, Dhaka 1229, Bangladesh; 3 Department of Statistics, University of Barishal, Barishal 8254, Bangladesh; 4 Department of Management, School of Business & Economics, North South University, Dhaka 1229, Bangladesh; 5 Department of Computer Science and Engineering (CSE), Rajshahi University of Engineering and Technology (RUET), Rajshahi 6203, Bangladesh; 6 Department of Mathematics and Statistics, Faculty of Science, Imam Mohammad Ibn Saud Islamic University (IMSIU), Riyadh 13318, Saudi Arabia; kazad@imamu.edu.sa (A.A.);; 7 Research Department, King Salman Center for Disability Research, Riyadh 11614, Saudi Arabia

**Keywords:** Parkinson’s disease, disability research, differential expression, protein–protein interaction network, key genes, drug repurposing, molecular dynamics simulation

## Abstract

**Background:** Parkinson’s disease (PD) is a complex neurodegenerative disorder in aged people with multifaceted molecular underpinnings. It poses a severe threat to millions of older adults worldwide. The understanding of the molecular mechanisms of PD development and the performance of its therapeutic strategies has not yet reached a satisfactory level. **Methods:** This study integrated six transcriptomic datasets to uncover key genes (KGs) and their underlying pathogenic mechanisms, providing insights into potential therapeutic strategies for PD. We designed a comprehensive computational pipeline using various bioinformatics tools and databases to investigate PD-causing KGs, focusing on their functions, pathways, regulatory mechanisms, and potential therapeutic drug molecules. **Results:** In order to explore PD-causing KGs, we initially identified 303 differentially expressed genes (DEGs) between PD and control samples with 204 upregulated and 99 downregulated DEGs using the LIMMA approach with threshold values at *Adj. p*-value < 0.05 and abs (log_2_FC) ≥ 1.0. Then, protein–protein interaction (PPI) network analysis pinpointed seven top-ranked DEGs (*GAPDH*, *PTEN*, *CCND1*, *APOE*, *ESR1*, *MAPK3/ERK1*, and *SNCA*) as KGs or central modulators of PD pathogenesis. Regulatory network analysis of KGs identified 3 top-ranked transcription factors (*FOXC1*, *NFKB1*, and *TFAP2A*) and 6 microRNAs (hsa-let-7b-5p, hsa-mir-16-5p, and others) as the pivotal regulators of KGs. Gene Ontology (GO) terms and KEGG pathway enrichment analyses with KGs revealed several crucial biological processes, molecular functions, cellular components, and neurodegenerative pathways associated with the development of PD. Finally, the top five molecules guided by KGs (Nilotinib, Bromocriptine, Withaferin-A, Celastrol, and Donepezil) were identified as promising drug candidates against PD and validated computationally through ADME/T analysis and molecular dynamics simulation studies. **Conclusions:** The findings of this study may serve as valuable resources for developing effective treatment strategies for PD patients.

## 1. Introduction

**Figure 1:**
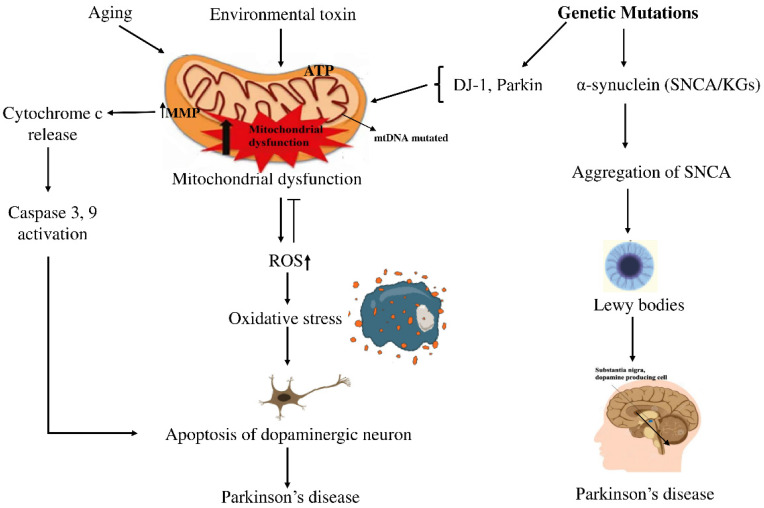
Schematic view of how genetic and environmental factors intertwine in PD. On one side, aging and exposure to certain toxins seem to push mitochondria into dysfunction, raising oxidative stress and activating caspases, steps that often end with dopaminergic neurons dying off. On the other side, mutations in *SNCA* drive α-synuclein clumping into LBs, the protein deposits long associated with PD. In the end, both routes point to the same outcome: the gradual loss of dopaminergic neurons, which remains the defining feature of the disease. Arrows (→) indicate the direction of biological processes or causal relationships between molecular events, while upward arrows (↑) denote an increase in activity or levels (e.g., ROS↑, MMP↑). Perpendicular lines (⟂) represent inhibition or blockage of specific cellular functions or pathways.

Parkinson’s disease (PD) is the second most common neurodegenerative disorder, characterized by the progressive degeneration of dopaminergic neurons in the substantia nigra (SN) among the elderly. This degeneration leads to a significant reduction in dopamine levels, resulting in the hallmark motor symptoms of PD, such as tremors, rigidity, and bradykinesia [1]. Currently, more than 10 million people are suffering from PD complexities worldwide [2], with projections estimating that the global PD population might rise to 12–17 million by 2040. Between 1999 and 2019, the number of deaths from PD increased significantly in all age groups, both men and women, people of different races and ethnicities, and in both urban and rural areas [3]. Treatment options from available medications focus only on reducing symptoms of PD [4]. Despite its widespread impact, there is no definitive treatment for it [5,6]. Therefore, more research is required to explore better treatments for PD. Historically, PD was predominantly attributed to environmental causes, but recent studies indicate that its development stems from a complex interplay between genetic factors and environmental influences [7,8]. This complex interplay contributes to a major pathological hallmark of PD, which is the formation of filamentous cytoplasmic inclusions, primarily composed of aggregated α-synuclein, which form Lewy bodies (LB) or Lewy neurites (LN). Phosphorylation and fibrillization of α-synuclein lead to LB formation and neuronal death [3]. Mitochondrial dysfunction caused by various harmful factors contributes to ROS production and oxidative stress in PD. The mtDNA mutations cause mitochondrial dysfunction by impairing the ETC, leading to ROS production. ROS caused the collapse of mitochondrial membrane potential (MMP) and disruption of complex I, leading to increased cytosolic Ca^2+^ and cytochrome c, which triggered apoptosis pathways [4]. A schematic diagram illustrating the relationship between genetic factors and PD is shown in Figure 1.

The precise mechanisms underlying PD remain complex, emphasizing the need to identify novel PD-causing genes that may offer valuable insights and potential targets for developing effective treatments and therapies [9]. Several individual studies, based on one or multiple datasets, have identified PD-causing key genes (KGs) through differential expression patterns and protein–protein interaction (PPI) analyses [10,11,12,13,14]. In these studies, authors identified differentially expressed genes (DEGs) between PD and control samples for each dataset individually. Then, they identified common DEGs (cDEGs) for multiple datasets to detect PD-causing KGs. However, their KG sets were not as consistent with each other. This may have occurred due to the identification of DEGs based on a small sample size, as most datasets consisted of a small sample size (*n* < 30). It is worth noting that statistical consistency depends on the sample size [15]. Therefore, this study aimed to explore more consistent PD-causing KGs by increasing the sample size in the dataset by merging multiple datasets after removing their batch effects in order to disclose drug targets and agents. To unravel the pathogenic mechanisms of the identified KGs, GO terms, KEGG-pathway enrichment analysis, and gene regulatory network (GRN) analysis were performed. Molecular docking analysis was carried out to identify potential drug candidates guided by KGs. The proposed drugs were further evaluated through ADME/T analysis to assess their pharmacokinetic and toxicological properties, followed by Molecular Dynamics (MD) simulations to investigate their stability, binding interactions, and dynamic behavior at the atomic level.

## 2. Methods and Materials

### 2.1. Data Acquisition

In order to explore PD-causing KGs and candidate drugs, we collected transcriptomics profiles datasets from the NCBI database and drug molecules through a literature review. We downloaded six microarray gene expression datasets from the GEO platform of the NCBI database with accession IDs GSE8397-GPL96 [16], GSE20141 [17], GSE49036 [18], GSE20163 [19], GSE20292 [20], and GSE20164 [21]. These datasets were generated from SN in the brain. Details of these datasets are provided in Table 1.

Furthermore, to investigate KG-guided candidate drug molecules for PD, 120 candidate molecules were collected from published articles and online databases, and then three-dimensional (3D) structures were retrieved from the PubChem database [22].

### 2.2. Integration of Transcriptomics Datasets and Identification of DEGs

The six datasets (CEL files) downloaded from GEO were processed using the Robust Multi-Array Average (RMA) algorithm [23]. Initial quality control involved removing low-expression genes, unannotated probe IDs, and non-overlapping probe sets between GPL96 and GPL570. All arrays were then normalized together using RMA, which performs background correction, quantile normalization, log_2_ transformation, and probe summarization based solely on the common probe set to minimize cross-platform bias. Dataset integration and batch harmonization were assessed using density plots, MA plots, and PCA before and after normalization (Figure S1). Following the approach of a previous study, the R (version 4.3.1) statistical package LIMMA (version 3.56.2) [24] was then applied to identify DEGs between PD and control samples. LIMMA employs a moderated *t*-test, which incorporates the square root of the moderated variance as the standard deviation [25]. DEGs between PD patient samples and healthy controls were significant at *Adj.-p*  <  0.05 and |log_2_ Fold Change| ≥ 1. Both log_2_-fold change (log_2_FC) and Adj. *p*-values were used as criteria to determine upregulated and downregulated DEGs. Probes lacking gene annotations were excluded. The probe exhibiting the highest log_2_FC was selected for genes represented by multiple probes.

### 2.3. Identification of KGs from DEGs

Proteins work through interactions with other proteins within the cell, and the PPI network analysis is utilized to identify KGs [26]. To construct the PPI network, the distance matrix ‘D’ is computed [27] as follows

$$D\;(i,\;j)=\frac{2|N_{i}\cap N_{j}|}{\left|N_{i}\right|+|N_{j}|}$$

where N*_i_* represents the set of neighbors for the *i*th protein, while N*_j_* denotes the neighbor set for the *j*th protein. A PPI network of DEGs was constructed using the STRING database [28] to pinpoint KGs. To extract significant KGs from this network, various topological metrics, including Degree, MNC, Edge Percolated Component (EPC), Closeness, Stress, and Radiality, were applied through the CytoHubba plugin within Cytoscape software (v3.10.0) [28]. The application of multi-parametric topological analysis enabled the detection of seven KGs, derived from the highest-ranked proteins following an extensive network assessment.

### 2.4. Validation of KG Expression Profiles and Their Association with PD

To independently confirm the expression patterns of the identified key genes in PD, box-plot analyses were performed using datasets retrieved from the NCBI database. Specifically, the GSE7621 dataset [29] was employed to assess differential expression between PD and control samples, and the resulting box plots verified the observed expression trends across both groups. To validate the association between PD and KGs, we applied the methodology from our earlier work [30,31], conducting disease-KGs enrichment analysis through the Enrichr tool [32] in combination with the DisGeNET database [33]. DisGeNET is a comprehensive database of gene–disease associations (GDAs) and variant–disease associations (VDAs). It uses a scoring formula to rank gene–disease associations. This scoring approach follows a probabilistic model that integrates evidence from multiple independent sources, producing a final value between 0 (indicating weak support) and 1 (indicating strong support).

### 2.5. Detection of Key Regulators of KGs

Transcription factors (TFs) and microRNAs (miRNAs) act as transcriptional and post-transcriptional regulators of protein-coding genes, respectively, within gene regulatory networks (GRNs). To identify primary TFs regulating KGs, we conducted a TF-KG interaction analysis using the JASPAR database [34] with the NetworkAnalyst platform [35]. Similarly, key miRNAs that impact KGs were identified through miRNA-KG interaction analysis using the TarBase database [36], facilitated by NetworkAnalyst. The most significant regulators were selected, and their interactions were visualized with Cytoscape. This comprehensive approach highlighted critical TFs and miRNAs that influence KGs at both transcriptional and post-transcriptional levels.

### 2.6. Detection of GO-Terms and KEGG-Pathways Associated with PD

The Gene Ontology (GO) framework categorizes gene functions into three domains: Molecular Function (MF), Cellular Component (CC), and Biological Process (BP) [37]. These categories are employed to describe the roles of KGs, focusing on their molecular mechanisms, cellular activities, and specific locations within the cell where they operate. Additionally, the Kyoto Encyclopedia of Genes and Genomes (KEGG) pathway database is commonly used to explore metabolic pathways and gene interactions [38]. To investigate the biological relevance of KGs, the Enrichr database web server [32] was utilized for conducting GO-term and KEGG pathway enrichment analyses. Also Gene Set Enrichment Analysis (GSEA) [39] was performed to identify significantly enriched KEGG pathways associated with the disease condition. Ranked gene expression data were analyzed using the GSEA tool, comparing “disease” versus “control” groups. Enrichment scores (ES) were calculated to assess the correlation of predefined gene sets with each phenotype, and the significance of enrichment was determined based on the position of pathway-related genes within the ranked dataset.

### 2.7. Exploring KGs-Guided Repurposable Drug Molecules Against PD

Drug discovery uses two main computational methods: de novo design, which creates new molecules but is time-consuming and costly, and drug repurposing, which tests approved drugs-protein interactions to find new therapies [40]. To explore KG-guided drug repurposing for PD, we collected 120 candidate molecules, as outlined in Section 2.1 of the method. In addition, we considered KGs-guided receptor proteins along with their regulatory transcription factor (TF) proteins for the docking study. The 3D receptor structures were sourced from the Protein Data Bank (PDB) [41], the AlphaFold Protein Structure Database [42], and the SWISS-MODEL database [43]. Discovery Studio Visualizer [44] was used to visualize the 3D structures of protein interactions. AutoDock tools (v1.2.3) were utilized for receptor protein preprocessing, which involved removing water molecules and adding charges. The drug molecules underwent energy minimization using Avogadro and were also preprocessed with AutoDock tools.

After preparing these, MD between receptors and ligands was then performed using AutoDock Vina [45] to calculate their binding affinity scores (kcal/mol). Let X_ab_ represent the binding affinity score between the ath receptor (*a* = 1, 2, …, *v*) and the bth ligand/agent (*b* = 1, 2, …, *u*). Receptors were organized in descending order according to their average binding scores;

$$\frac{1}{v}\sum_{b=1}^{u}X_{ab};\;a=1,\;2,\;\ldots ,v$$

and ligands/agents were similarly ranked based on their average scores in descending order as follows

$$\frac{1}{u}\sum_{a=1}^{v}X_{ab};\;b=1,\;2,\;\ldots ,u$$

This ranking process was utilized to identify the highest-scoring ligands/agents as potential drug candidates.

### 2.8. In Silico Validation of Top-Ranked Drug Molecules

#### 2.8.1. ADME/T Analysis

ADME/T analysis appraises the absorption, distribution, metabolism, excretion, and toxicity of drug candidates, providing essential insights into their pharmacokinetic and safety profiles. This analysis is vital for the early identification of potential safety issues, guiding drug design and dosage optimization, and supporting regulatory approval by confirming the drug’s safety and efficacy. Therefore, the top six ranked drug compounds were evaluated for their drug-like characteristics and ADME/T profiles to gain deeper insight into their structural attributes and chemical properties. Compliance with Lipinski’s rule was assessed through the SCFBio web application [46]. Following this, ADME/T properties were predicted using the SwissADME database [47] and pkCSM databases [48]. These predictions were based on the most favorable drug compound structures, represented in SMILES format, to ensure accurate calculations.

#### 2.8.2. Molecular Dynamics (MD) Simulations

MD simulations were performed using YASARA dynamics software (version 22.8.22.W.64) [49] with the AMBER14 force field [50] to explore the dynamic behavior and stability of the top protein–ligand complexes. Six distinct systems were utilized to run MD. The top three leading interactions included *MAPK3*_Nilotinib, *PTEN*_Withaferin-A, and *GAPDH*_Bromocriptine, corresponding to our candidate receptors. Prior to the simulation, the hydrogen bond networks of the selected complexes were refined and immersed in a TIP3P water model. Solvent density was calibrated to 0.997 g/mL to ensure periodic boundary conditions. An initial energy minimization was performed using the steepest descent method over 5000 cycles. Simulations were conducted under standard physiological conditions (298 K, pH 7.4, 0.9% NaCl) and utilized a multi-time-step algorithm [51], incorporating a 2.50 femtosecond (fs) time-step interval [52]. A 100-nanosecond MD was carried out using a Berendsen thermostat [53] to regulate temperature and maintain constant pressure, ensuring a stable and realistic environment for the simulation. Simulation trajectories were recorded at 250-picosecond (ps) intervals, offering detailed snapshots of system behavior for comprehensive, in-depth analysis. The initial study was performed using the default script of the YASARA macro and SciDAVis (https://scidavis.sourceforge.net). Subsequently, binding free energy calculations based on MM-PBSA (MM-Poisson–Boltzmann surface aolecular Mechanics–Poisson–Boltzmann Surface area) were carried out with YASARA software (version 22.8.22.W.64), applying the following formula to compute the binding free energy [54].

Binding Free Energy = E_potReceptor_ + E_solvReceptor_ + E_solvLigand_ + E_potLigand_ − E_solvComplex_ − E_potComplex_

The binding energies were determined through MM-PBSA analysis utilizing YASARA’s built-in macros with the AMBER 14 force field, where more positive values indicate stronger binding [55].

### 2.9. The Workflow of the Study

**Figure 2:**
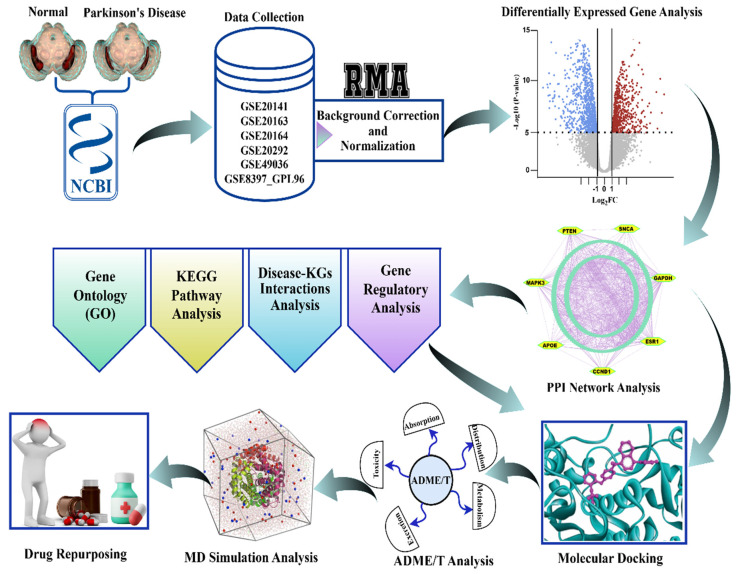
The graphical workflow for the identification of biomarker genes and therapeutic agents for Parkinson’s disease. Normalized and corrected transcriptomic data downloaded from NCBI GEO underwent differential expression analysis. Significant genes went through GO, KEGG, disease-KG, and regulatory network analysis, after which PPI network analysis was conducted to identify the hub genes. Prospective drugs went through molecular docking, ADME/T, and MD simulation, resulting in candidate repurposing therapies for Parkinson’s disease.

The workflow of this study, as described in Section 2.1, Section 2.2, Section 2.3, Section 2.4, Section 2.5, Section 2.6, Section 2.7, Section 2.8 and Section 2.9, is graphically illustrated in Figure 2 for convenience of presentation.

## 3. Result

### 3.1. Quality Control and Batch Effect Integration

To ensure the reliability of the integrated gene expression dataset, we employed a rigorous quality control and batch correction workflow. The first steps focused on removing technical noise and reducing systematic bias present in the raw data. As expected, the unprocessed expression values (Figure S1A) exhibited a pronounced right-skewed distribution, a common feature in raw microarray data, where many genes display low signal intensity. Following ${log}_{2}$ transformation and normalization, the distribution was successfully centered and became more symmetrical (Figure S1B), confirming that the data are appropriately scaled for downstream statistical methods.

Principal Component Analysis (PCA) was used to visualize the dominant sources of variation before and after integration. PCA of the combined, normalized data revealed that the primary source of variance was the technical batch effect (GSE Study ID). Samples clustered tightly and distinctly based on their original batch (color), with PC1 explaining a high percentage of the total variance (86.0%) (Figure S1C). This confirmed that the batch effect was strong enough to completely mask any underlying biological signal. After applying the batch correction, the PCA plot showed that the batch-specific clustering was eliminated (Figure S1D). Samples from different batches (colors) became well-intermingled and overlapping (Figure S1D). The explained variance was more evenly distributed across the principal components, signifying that the most significant remaining sources of variation were now related to biological factors, such as the differences between the Control and Disease groups.

We assessed intensity-dependent bias by comparing a representative pair of samples. The MA plot for the pre-normalized data (Figure S1E) showed a clear curved trend and high variance (fanning) at low average expression values, indicating a strong systematic bias. Following normalization, the data were successfully aligned to the horizontal M = 0 axis (Figure S1F), demonstrating the effective removal of this bias and establishing data comparability across the intensity spectrum.

### 3.2. Differential Gene Expression Patterns Identified from Integrated Transcriptomes

At first, the CEL file formats of the six datasets were downloaded from the GEO platform of the NCBI database. Then, their batch effects (background correction) were removed and normalized using the robust multi-array averaging (RMA) algorithm. Afterward, these datasets were combined, comprising 79 patients with PD and 63 age-matched controls, as shown in Table 1. Then, we performed differential expression analysis by applying the statistical LIMMA technique and identified 303 DEGs that exhibited significant alterations in PD compared to controls (*Adj. p*-value < 0.05 & |log_2_FC| ≥ 1). Among these DEGs, 204 demonstrated upregulation, while 99 showed downregulation in PD samples (Table S1), suggesting substantial transcriptional remodeling in the disease state.

### 3.3. Key Genes Identified from Differentially Expressed Gene Analysis

**Figure 3:**
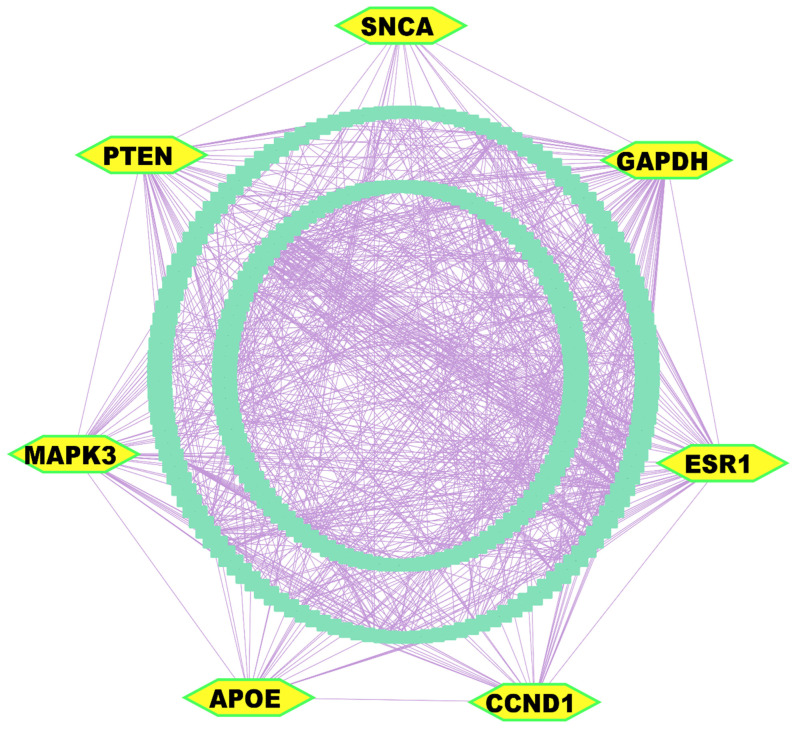
PPI network analysis of DEGs revealed KGs, highlighted as yellow nodes within the interactome network.

The PPI network analysis was conducted utilizing the STRING database [56], with 303 DEGs. Implementation of multi-parametric topological analytics facilitated the identification of seven KGs. These pivotal molecular modulators (*GAPDH*, *PTEN*, *CCND1*, *APOE*, *ESR1*, *MAPK3/ERK1*, and *SNCA*) appeared as central orchestrators within the constructed interactome network, of which *GAPDH*, *CCND1*, and *ESR1* were upregulated; in contrast, *SNCA*, *PTEN*, *APOE*, and *MAPK3* were downregulated (Figure 3 and Table S2).

### 3.4. Regulatory Network Analysis Reveals Key Regulators of Key Genes

**Figure 4:**
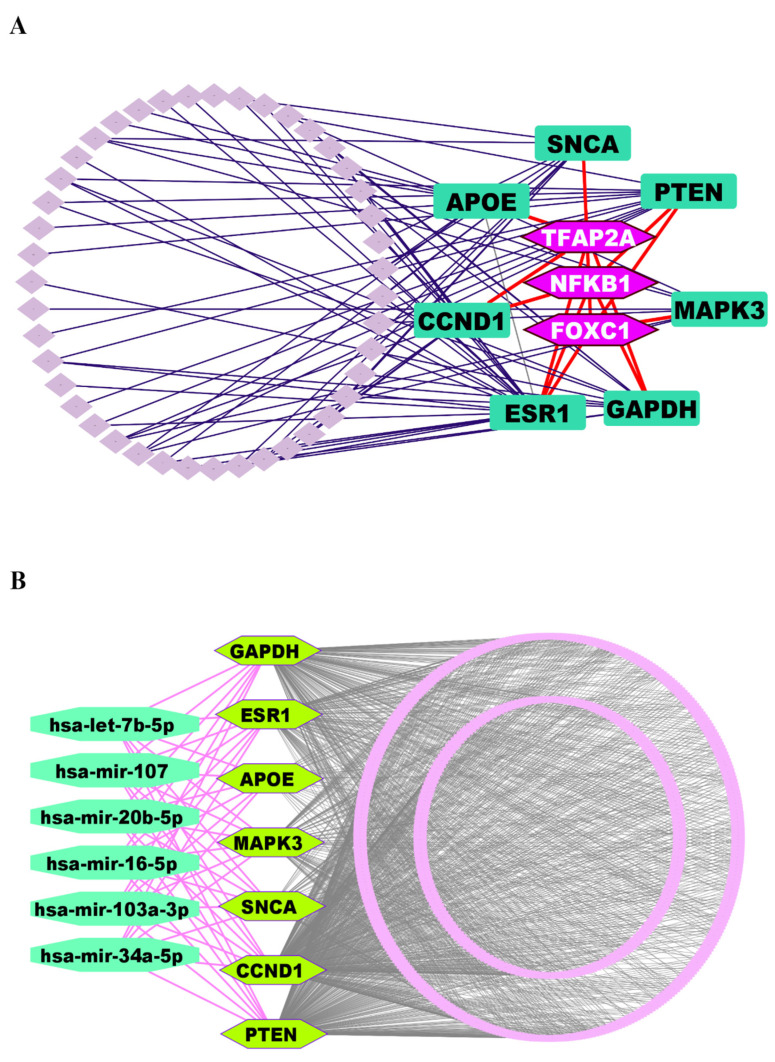
(**A**) TFs-KGs interactome network to explore key regulatory factors of KGs, where KGs are ornamented as round rectangle-shaped markers in dark green color, and TFs are highlighted in hexagon-shaped markers with light purple color. (**B**) The miRNA-KGs interactome network, where KGs are depicted as hexagon-shaped markers in pure green color, and miRNAs are displayed as round octagon markers in lime green.

We investigated the regulatory networks involving TFs and miRNAs that govern the expression of the identified KGs. This analysis aimed to uncover both the transcriptional and post-transcriptional mechanisms controlling the KGs. For the transcriptional regulators, we selected the top three TFs, such as *FOXC1*, *NFKB1*, and *TFAP2A*, based on their high values for the topological metrics of betweenness ≥ 85.60 and degree ≥ 4 within the regulatory network (Figure 4A).

Similarly, to identify the post-transcriptional regulators, we applied the same topological criteria, this time using a degree cutoff of ≥7 and a betweenness cutoff of ≥588.89. This led us to select the top six miRNAs (hsa-mir-16-5p, hsa-mir-34a-5p, hsa-let-7b-5p, hsa-mir-103a-3p, hsa-mir-107, and hsa-mir-20b-5p) as key regulators of the KGs at the post-transcriptional level (Figure 4B).

### 3.5. KGs Validation of KG Expression Profiles and Their Association with PD

We validated the differential expression of the KGs using an independent dataset, comprising 16 PD and 9 control samples, and visualized the results through box-plot analysis (Figure S2). This assessment confirmed our initial observations, showing that three KGs were upregulated and four were downregulated in PD. Disease-gene association analysis was performed by querying the DisGeNET database within the Enrichr platform [31]. This revealed that the identified KGs exhibited statistically significant relationships (*Adj. p*-value < 0.05) with several prominent neurological disorders (Alzheimer’s disease, Brain neoplasms, Motor symptoms, Neuroblastoma, Astrocytoma), including PD. These findings suggest the centrality of the detected KGs in the pathogenesis of Parkinson’s and related neuronal diseases (Table S3).

### 3.6. Functional Enrichment Analysis Reveals GO Terms and KEGG Pathways Associated with PD

**Figure 5:**
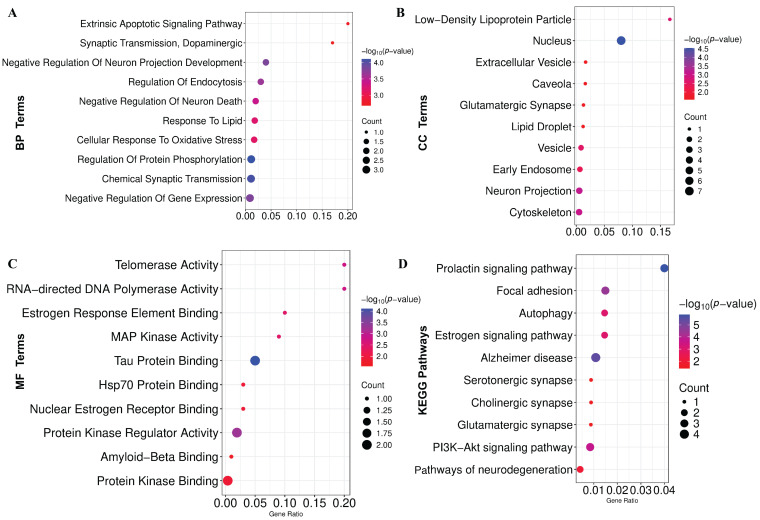
Functional enrichment of KGs. (**A**) Biological process (BP). (**B**) Cellular component (CC). (**C**) Molecular function (MF). (**D**) KEGG pathway enrichment analysis.

GO-terms and KEGG-pathway enrichment analyses were conducted on the KGs to gain comprehensive mechanistic insights into the underlying pathobiology of PD. The significantly enriched BPs, MFs, and CCs are demonstrated in Figure 5A–C and Table S4, respectively. Additionally, the considerably overrepresented KEGG pathway is illustrated in Figure 5D and Table S4. Also, we have conducted GSEA that revealed significant enrichment of pathways associated with neurodegenerative and signaling processes (Figure S3).

### 3.7. Exploring KGs-Guided Drug Molecules by Molecular Docking Analysis

**Figure 6:**
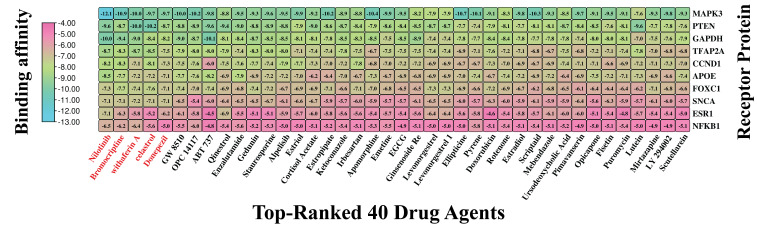
Molecular docking outcomes display the highest-ranking 40 drug candidates aligned with their specific receptors. Binding scores are represented, where sky hues denote strong interactions between the drug compounds and target proteins, while pink hues signify weaker affinities. Also, a larger negative value indicates a stronger binding affinity. This visual representation places the top 40 drug agents (out of 120) on the *X*-axis and the 10 proposed target proteins along the *Y*-axis. Drug names highlighted in red indicate the top five compounds with the strongest binding affinities across multiple receptor proteins.

For selecting top-ranked candidate drug molecules by molecular docking analysis, as introduced in Section 2.7, we considered 120 candidate drug compounds (ligands) (Table S5) and ten target proteins (receptors) derived from our identified seven KGs and their three principal transcriptional regulators. To do so, 3D structures of 9 receptors (*GAPDH*, *PTEN*, *CCND1*, *APOE*, *ESR1*, *MAPK3*, *SNCA*, *NFKB1*, and *TFAP2A*) were downloaded from the Protein Data Bank (PDB) with source IDs 4WNC, 5BZZ, 6P8E, 2KC3, 5T0X, 4QTB, 1XQ8, 1MDI, and 8J0L, respectively. The remaining one (*FOXC1*) was downloaded from AlphaFold with the UniProt ID Q12948 as the source. On the other hand, 120 ligands with 3D structures were taken out from the PubChem database [22], as previously noted. Following this, a molecular docking analysis was conducted to assess the binding interactions between 10 target receptors and 120 drug compounds. The resulting BA scores, expressed in kcal/mol, were organized into a binding matrix M = (Mij). Ordinal ranking was established by arranging receptors and drug agents in descending order based on their respective row and column sums of the BA matrix (Figure 6 and Table S6). Nilotinib emerged as the top lead compound, demonstrating strong binding affinity (BAS < −6.5 kcal/mol) with all 10 target receptor proteins. Additionally, four other leading candidates (Bromocriptine, Withaferin-A, Celastrol, and Donepezil) also showed significant binding (BAS < −6.5 kcal/mol) with 8 out of 10 receptors. Based on these results, these five drugs were identified as promising therapeutic candidates for PD. Detailed interaction profiles between the top three receptor targets and compounds are provided in Table S7, suggesting their potential therapeutic efficacy.

### 3.8. In Silico Validation Confirms the Potential of Top-Ranked Drug Molecules

#### 3.8.1. Pharmacokinetic Analysis

The ADME profile of a drug molecule is fundamental to defining its pharmacokinetic properties and overall therapeutic performance. A detailed analysis of these properties is essential to confirm that the drug achieves high bioavailability, distributes adequately across target tissues, follows well-defined metabolic pathways, and is effectively eliminated from the body. The ADME/T characteristics of the five drugs were assessed using different parameters. Withaferin-A and Donepezil fully adhered to Lipinski’s rule [57], confirming their drug-like properties, whereas Nilotinib, Bromocriptine, and Celastrol each exhibited a single rule violation. The lipophilicity (LogP values) of these five compounds aligns with Lipinski’s range (1 to ≤5), confirming their classification as lipophilic agents except Celastrol (Table 2). Several characteristics can be used to assess their toxicity and ADME. These compounds show potential as oral drug candidates due to their predicted high gastrointestinal absorption. A compound is generally regarded as well absorbed in the human intestines when its Human Intestinal Absorption (HIA) score exceeds 30% [48]. In our analysis, all five proposed drugs demonstrated robust absorption properties, each with an HIA score of ≥71%, indicating their strong potential for absorption in the human body. Blood–brain barrier (BBB) penetration is a critical determinant for therapeutic efficacy in neurological disorders.

The BBB permeability index, expressed as logBB, quantitatively predicts a compound’s ability to traverse this physiological interface between systemic circulation and the central nervous system. Compounds exhibiting logBB values exceeding 0.3 demonstrate favorable BBB penetration, whereas those with logBB below −1 show limited brain distribution [58,59]. Given that our investigation focuses on PD, a progressive neurodegenerative disorder, BBB penetrance represents a crucial parameter for therapeutic efficacy, as candidate compounds must effectively traverse this barrier to reach their intended molecular targets within the brain parenchyma. The five drug candidates proposed in our study demonstrate a strong potential for BBB penetration (Table 3), suggesting their suitability for central nervous system activity. Toxicity assessments of our proposed compounds, including AMES testing, LD50 analysis, and minnow LC50 evaluation, demonstrated non-toxicity across almost all parameters. These findings suggest that the compounds exhibit drug-like characteristics and are suitable for oral administration.

#### 3.8.2. MD Simulations Validate the Binding Stability of Selected Drug Molecules

**Figure 7:**
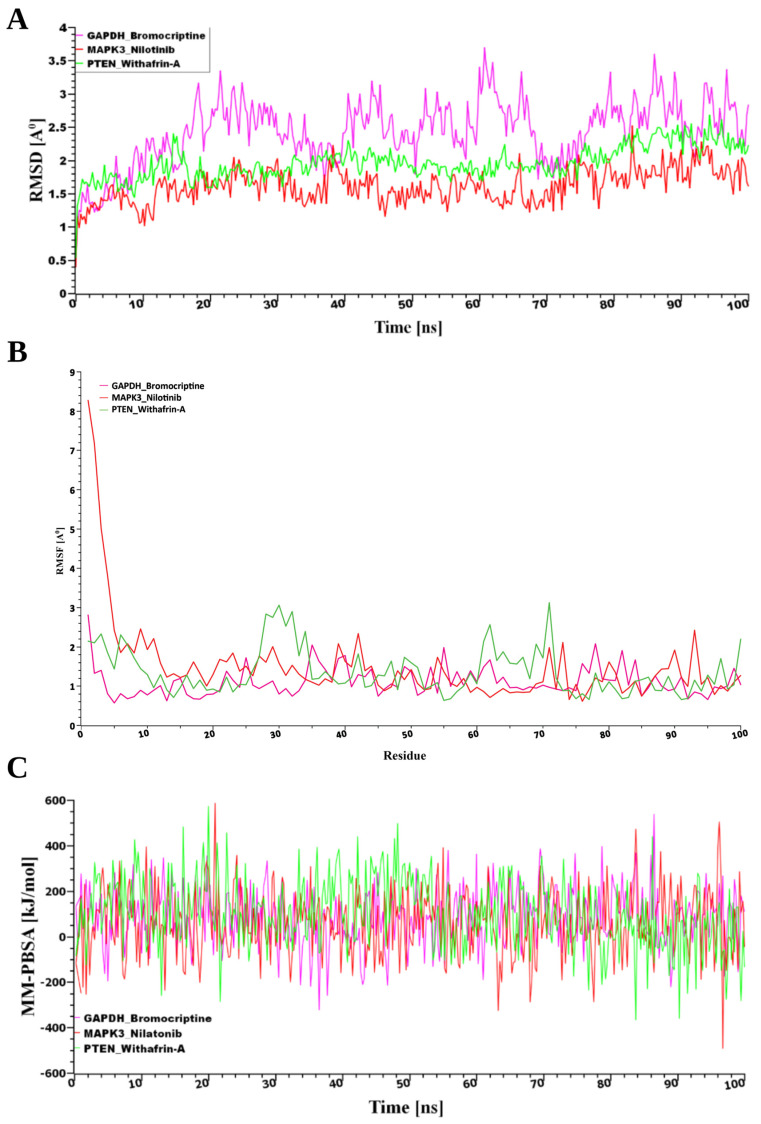
(**A**) Time-dependent analysis of the RMSDs of the backbone atoms (C, Cα, and N) was conducted for each protein–ligand docked complex, (**B**) The root mean square fluctuation (RMSF), and (**C**) The binding free energy (expressed in kJ mol−1) for each simulation snapshot was assessed using the MM-PBSA method, indicating variations in binding stability throughout the simulation; higher values denote stronger binding interactions. The protein complexes are represented as follows: pink for *GAPDH*, green for *PTEN*, and red for *MAPK3*.

From the pool of potential therapeutic compounds, the top three were Nilotinib, Bromocriptine, and Withaferin-A (Figure 7). Consequently, these top three drugs were chosen for stability analysis using 100 ns MD-based MM-PBSA simulations. From Figure 7, our study revealed that all three systems maintained remarkable stability despite variations in both the movement and the initial configurations of the drug–target complexes. The root mean square deviation (RMSD) was computed to assess this. Figure 7A illustrates the RMSD values corresponding to the selected receptors, including *PTEN*, *MAPK3*, and *GAPDH*. Despite structural variations observed between the initial and dynamic drug-target complexes, all three systems demonstrated notable stability. The stability was evaluated using RMSD analysis, as shown in Figure 7A. For the predicted receptor complexes (*MAPK3*, *PTEN*, and *GAPDH*), RMSD values generally fell within a range of ~1 Å to 2.5 Å, with the exception of *GAPDH*, which exhibited a broader range of ~2 Å to 3.7 Å. The average RMSD values for the *MAPK3*, *PTEN*, and *GAPDH* complexes were 1.62 Å, 1.96 Å, and 2.43 Å, respectively. Among these, the *MAPK3* complex exhibited a more rigid conformation compared to the other proteins. In contrast, the *GAPDH* complex demonstrated greater flexibility, with RMSD values gradually increasing from 2 Å to 3.7 Å throughout the simulation. By contrast, RMSF analysis, reflecting residue-level flexibility, revealed that the *GAPDH*–Bromocriptine complex exhibited the greatest structural stability, with a mean value of 1.1168 Å compared to 1.3679 Å for *PTEN*–Withaferin-A and 1.5046 Å for *MAPK3*–Nilotinib, indicating more consistent target interactions (Figure 7B).

Furthermore, analysis of binding energies using MM-PBSA calculations revealed that *PTEN* had the strongest interaction with the drug (102.85 kJ/mol), followed by *GAPDH* (85.16 kJ/mol) and *MAPK3* (59.18 kJ/mol), as shown in Figure 7B. This suggests that while *MAPK3* maintained the most stable structural conformation, *PTEN* formed the most energetically favorable complex with the drug molecule.

## 4. Discussion

PD is a lifelong, progressive neurodegenerative disorder with no known cure, gradually worsening over time [60,61]. Despite extensive research, the precise molecular mechanisms underlying PD remain elusive [62], necessitating advanced bioinformatics analysis to elucidate pathogenetic mechanisms, therapeutic targets, and agents. In this study, we combined and analyzed six transcriptomics datasets using the LIMMA statistical framework and identified 303 DEGs between the PD and control groups. The PPI network analysis revealed seven DEGs as the KGs of PD pathogenesis. A disease–gene association study based on the DisGeNET database also revealed that KGs are significantly associated with various neurological disorders, including PD. The KGs-TFs and KGs-miRNAs interaction network analysis revealed three top-ranked TF proteins and six miRNAs that are associated with the transcriptional and post-transcriptional regulation of KGs. Functional enrichment analyses of KGs with GO-terms and KEGG pathways revealed some crucial biological processes (Chemical Synaptic Transmission, Negative Regulation Of Neuron Death, Cellular Response To Oxidative Stress, Positive Regulation Of Extrinsic Apoptotic Signaling, and Dopaminergic), molecular functions (Tau Protein Binding, Nuclear Estrogen Receptor Binding, Hsp70 Protein Binding), cellular components (Nucleus, Neuron Projection, Cytoskeleton, Extracellular Vesicle, and Glutamatergic Synapse), and KEGG-pathways (AD, PI3K-Akt signaling pathway, Autophagy, Pathways of neurodegeneration) that are associated with the PD pathogenesis.

### 4.1. KGs, Regulatory Networks, and Pathways in PD Pathogenesis

Importantly, most of the identified KGs are supported by prior PD studies and appear in PD-curated databases (DisGeNET), suggesting that their prioritization reflects genuine biological involvement rather than generic network centrality. Among PD-causing KGs, *GAPDH* (glyceraldehyde-3-phosphate dehydrogenase) is located on chromosome 12p13. The rs1136666 CC allele polymorphisms of *GAPDH* indicate a high risk of PD [63]. The S-nitrosylation of *GAPDH* under oxidative stress can trigger neuronal damage through nuclear translocation. It interacts with α-synuclein and other proteins implicated in neurodegenerative disorders [64,65,66]. This study revealed that *NFKB1* and *TFAP2A* regulate the expression of *GAPDH*. Many researchers have reported that *NFKB1*, which encodes NF-κB, serves as a key transcription factor regulating the expression of inflammatory genes and innate immune responses [67,68].

*Cyclin D1* (*CCND1*) is a key regulator of cell cycle progression driven by extracellular cues, and its abnormal expression has been associated with both tumorigenesis and apoptosis in terminally differentiated neurons [69,70]. Recent studies have further shown elevated *CCND1* levels in PD cell models, linking its upregulation to α-synuclein-induced neurotoxicity. Conversely, silencing *CCND1* has been demonstrated to protect against neuronal death [71].

The *PTEN*, governed by the transcriptional regulation of *FOXC1* and *NFKB1*, is a major cause of neuronal cell death and may be a biological target for creating innovative treatment approaches for PD [72]. Its involvement in the *autophagy pathway* and increased oxidative stress contribute to neuronal apoptosis, leading to the loss of dopaminergic neurons in the SN, a key feature of PD [73]. Numerous research studies have highlighted that Apolipoprotein E (*ApoE*) is a key determinant of vulnerability in PD, predominantly secreted by astrocytes, and the most abundant apolipoprotein in cerebrospinal fluid (CSF) [74,75].

Enrichment analysis revealed a strong association between the regulation of *nervous system development* and cellular components, including endocytic vesicles and early endosomes. miRNA (*hsa-let-7b-5p*) was identified as a key post-regulator of *ApoE*, with evidence suggesting that it functions as a critical regulator of developmental pathways and has been linked to the pathogenesis of PD [76]. The overexpressed estrogen receptor-α (*ESR1*) gene encodes a protein associated with neuroinflammation [77], which is regulated by transcription factors including *FOXC1*, *NFKB1*, and *TFAP2A*. Neuroinflammation contributes to BBB disruption, enabling immune cells and toxins to enter the brain. This exacerbates the inflammatory response and accelerates neuronal damage [78].

The *MAPK3* also known as *ERK1* gene, which exhibits reduced expression in PD, is regulated by the transcription factor *FOXC1* and plays a crucial role in the pathogenesis of the disease. An in vivo study demonstrated that nootkatone effectively suppresses *MAPK3* expression by activating the *PI3K/Akt* signaling pathway, thereby alleviating neuroinflammation and ameliorating symptoms in a rotenone-induced PD model [79,80]. The *SNCA* gene, situated on chromosome 4, encodes the alpha-synuclein protein [81], a protein whose genetic variants are strongly associated with an increased risk of developing PD [82,83]. Additionally, the tau protein and *SNCA* risk genotypes showed a marginal association with PD susceptibility individually; however, their combined effect nearly doubled the risk of developing PD [84]. Tau aggregation, driven by post-translational modifications, disrupts microtubule networks, affects tau protein binding, and contributes to neuronal degeneration in PD [85].

### 4.2. Drug Discovery and Therapeutic Insights

#### 4.2.1. New In Silico Results

Molecular docking analysis identified five top-ranked drug molecules as potential inhibitors of *PTEN*, *CCND1*, *ESR1*, and *SNCA*, and as activators of *GAPDH*, *APOE*, and *MAPK3*. To assess the viability of the identified drug candidates, we conducted drug-likeness evaluations along with comprehensive ADMET profiling. The drug-likeness assessment, guided by Lipinski’s Rule of Five, examined key pharmacokinetic parameters, including molar refractivity, log P, hydrogen bond donors, hydrogen bond acceptors, and molecular mass. All compounds were deemed drug-like, meeting at least four of Lipinski’s criteria. Additionally, the selected molecules exhibited favorable ADMET profiles, including sufficient water solubility, high Human Intestinal Absorption (HIA) levels ranging from 71.35% to 99.53%, and no observed carcinogenic effects. Finally, to assess the stability of the three highest-ranked compounds (Bromocriptine, Nilotinib, and Withaferin-A), MD simulations combined with MM-PBSA were conducted using 100 nanoseconds. These simulations were performed on the top three proposed receptor targets (*GAPDH*, *MAPK3*, and *PTEN*). The results demonstrated that the interactions of these drugs with the receptors were consistent and adhered to established physical principles [86].

It should be noted that all of our predicted drug molecules, Nilotinib [1,87], Bromocriptine [88,89], Withaferin-A [90,91], Celastrol [92,93], and Donepezil [94,95], have been validated experimentally as promising therapeutic options for PD in previous independent studies. As highlighted in the referenced article, Nilotinib has the potential to enhance dopamine metabolism, offering a therapeutic avenue for addressing both motor and nonmotor symptoms of PD. In vivo studies suggest that Bromocriptine holds significant potential as a novel therapeutic agent for PD. Notably, at a mean dose of 57 mg, Bromocriptine demonstrated a statistically significant improvement in rigidity, tremor, bradykinesia, gait disturbances, and overall clinical scores [88]. *Withania somnifera* (L.) Dunal, known as “Ashwagandha” in Sanskrit, is rich in bioactive compounds such as withaferin-A and withanolide-A. Emerging evidence from preclinical research and select clinical trials underscores its neuroprotective potential in combating neurodegenerative diseases like Parkinson’s, Alzheimer’s, and Huntington’s disease. Additionally, it alleviates apoptosis, inflammation, and oxidative stress processes [91]. Celastrol shows potential for preventing and treating PD by promoting mitophagy, which clears dysfunctional mitochondria and thereby prevents dopaminergic neuronal apoptosis, offering neuroprotection. Interestingly, in mice, it alleviates motor impairments, reduces neurodegeneration in the substantia nigra and striatum, and enhances striatal mitophagy [92,93].

#### 4.2.2. A Previously Validated Drug

On top of that, Donepezil, also known as Aricept, is FDA-approved for treating Parkinson’s, Alzheimer’s, and other forms of dementia, as listed in the DrugBank database (DB00843). Mori et al. reported that Donepezil demonstrates significant efficacy in treating PD with dementia, leading to remarkable clinical improvements [94,95]. Therefore, the findings of this study might be valuable resources for taking an effective treatment plan against PD.

### 4.3. Limitations and Future Direction

This study, while aiming to mitigate dataset heterogeneity by merging multiple transcriptomic datasets and mitigate batch effects, still faces certain constraints. A major limitation is the incomplete and non-uniform metadata across the six GEO cohorts, which prevented the creation of a harmonized cohort-level table and limited the ability to perform covariate adjustment. Additionally, although cross-platform normalization helped reduce technical variation, the use of microarray-based data inherently carries less resolution and dynamic range compared to newer sequencing technologies. While in silico molecular docking and molecular dynamics simulations provided valuable insights into potential drug–target interactions, their predictive accuracy remains limited by the computational models and force fields employed. Therefore, experimental validation through in vitro and in vivo studies, as well as future analyses using larger and more diverse RNA-seq datasets, will be essential to confirm and extend these findings. Despite these limitations, the study offers robust bioinformatic evidence and a solid foundation for developing and validating novel therapeutic strategies for PD.

## 5. Conclusions

This study provides significant insights into the molecular underpinnings of PD through the identification of 303 DEGs, including KGs such as *GAPDH*, *PTEN*, *CCND1*, *APOE*, *ESR1*, *MAPK3*, and *SNCA*. The GO enrichment analysis revealed crucial BP, MF, and CC associated with PD, enhancing our understanding of its pathobiology. Additionally, KEGG-pathway analysis identified significantly overrepresented pathways that are pivotal in neurodegenerative processes, further elucidating the molecular mechanisms involved in PD. The regulatory network analysis identified the top three transcription factors (*FOXC1*, *NFKB1*, and *TFAP2A*) and six microRNAs (*hsa-mir-16-5p*, *hsa-mir-34a-5p*, *hsa-let-7b-5p*, *hsa-mir-103a-3p*, *hsa-mir-107*, and *hsa-mir-20b-5p*) that modulate these key genes, suggesting potential targets for therapeutic intervention. Finally, KGs-guided top-ranked five drug molecules (Nilotinib, Bromocriptine, Withaferin-A, Celastrol, and Donepezil) were recommended for PD, where Bromocriptine (DB01200) is an already-FDA-approved drug for hyperprolactinemia-related conditions and early Parkinson’s disease; Donepezil (DB00843) is approved for cognitive and behavioral symptoms in Alzheimer’s, Parkinson’s, and other dementias; Nilotinib (DB04868) is approved for treating Philadelphia chromosome-positive Chronic Myeloid Leukemia (CML), including imatinib-resistant cases; Withaferin-A, which is not yet approved for any diseases but a natural compound from Withania somnifera (Ashwagandha) with neuroprotective potential for PD; and Celastrol (DB18736), which is not yet FDA-approved but under investigation for cancer, metabolic, and neurodegenerative diseases. However, some of these drug molecules may require further experimental validation to confirm their therapeutic efficacy for PD.

## Figures and Tables

**Table 1:** Summary of gene expression datasets used for PD analysis. The table presents details of microarray experiments, including dataset IDs (GEO accession numbers), study platforms, sample sizes (Control vs. PD), and Country.

GEO Accession	Platform	Sample Size		Country
		Control	Disease	
GSE20141	GPL570	8	10	USA
GSE49036	GPL570	8	20	Netherlands
GSE20164	GPL96	5	6	USA
GSE20292	GPL96	18	11	USA
GSE20163	GPL96	9	8	USA
GSE8397	GPL96	15	24	United Kingdom
Total		63	79	

**Table 2:** Drug likeness profile of candidate drug molecules.

Lipinski Rule		Surface Area	H-Bond Donor (HBD)	H-Bond Acceptor (HBA)	LogP	Molecular Weight	Compounds
Violation	Follow						
1	3	221.175	2	7	4.153	529.18	Nilotinib
1	3	259.451	3	6	3.1928	654.606	Bromocriptine
0	4	201.317	2	6	3.3529	470.6	Withaferin A
1	3	197.082	2	3	6.6977	450.61	Celastrol
0	4	167.005	0	4	4.3611	379.5	Donepezil

**Table 3:** ADME and Toxicity (ADME/T) profile of the 5 top-ranked drug molecules.

Toxicity			Excretion	Metabolism	Distribution		Absorption		Compounds
LD50 (mole/kg)	LC50 (log mM)	AMES	TC	CYP3A4 Inhibitor	CNS LogPS	BBB (LogBB)	P-gpI	HIA (%)	
					(Permeability)				
2.489	1.301	No	0.406	Yes	−2.052	−0.684	Yes	99.538	Nilotinib
3.739	2.448	No	0.327	Yes	−2.601	−0.711	Yes	71.357	Bromocriptine
2.779	0.738	No	0.435	No	−2.72	−0.03	Yes	85.345	Withaferin A
2.362	−0.642	No	−0.094	Yes	−1.278	0.078	No	100	Celastrol
2.753	−2.011	No	0.987	Yes	−1.464	0.157	Yes	93.707	Donepezil

## Data Availability

All transcriptomics data used in this study were obtained from the Gene Expression Omnibus (GEO) database (https://www.ncbi.nlm.nih.gov/geo/) (accessed on 19 April 2025). of the National Center for Biotechnology Information (NCBI) through the following code: GSE8397-GPL96, GSE20141, GSE49036, GSE20163, GSE20292, and GSE20164.

## Supplementary Materials

The following supporting information can be downloaded at: https://www.mdpi.com/article/10.3390/genes16121459/s1, Figure S1. Pre- and Post-Integration Quality Control, MA plot and PCA visualization. Figure S2. Expression patterns of KGs with Box-plots for Parkinson’s disease. Figure S3. Gene Set Enrichment Analysis (GSEA) plots. Table S1. List of upregulated and downregulated DEGs in PD from 6 datasets (GSE8397-GPL96, GSE20141, GSE49036, GSE20163, GSE20292, and GSE20164); Table S2. List of key genes (KGs) from PPI network based on different topological measures; Table S3. Verification of association between PD and KGs; Table S4. List of GO and KEGG terms based on Key Genes; Table S5. Collection of PD related candidate drug agents from published articles and others sources; Table S6. Docking/(binding affinity) scores (kcal/mol) between the proposed target genes/proteins (receptors) and top ordered 40 candidate drugs (out of 120); Table S7. The 3-dimension view of strong binding interactions between targets and drugs. References [96,97,98,99,100,101,102,103,104,105] are cited in the supplementary materials.

## Abbreviations

The following abbreviations are used in this manuscript:

PD	Parkinson’s Disease
AD	Alzheimer’s Disease
SN	Substantia Nigra
LB	Lewy Bodies
LN	Lewy Neurites
MMP	Mitochondrial Membrane Potential
ROS	Reactive Oxygen Species
RMA	Robust Multi-Array Average
KGs	Key Genes
DEGs	Differentially Expressed Genes
cDEGs	Common Differentially Expressed Genes
GRN	Gene Regulatory Network
PPI	Protein–Protein Interaction
GDAs	Gene–Disease Associations
VDAs	Variant–Disease Associations
TFs	Transcription Factors
miRNAs	microRNAs
GO	Gene Ontology
KEGG	Kyoto Encyclopedia of Genes and Genomes
PDB	Protein Data Bank
BA	Binding Affinity
MM-PBSA	MM–Poisson–Boltzmann Surface Area
MD	Molecular Dynamics
ADME	Absorption, Distribution, Metabolism, and Excretion
BBB	Blood-Brain Barrier
