# Supplementary material for: Multi-Transcriptome-Informed Network Pharmacology Reveals Novel Biomarkers and Therapeutic Candidates for Parkinson’s Disease
Source: Genes (Basel). 2025 Dec 7;16(12):1459. doi: 10.3390/genes16121459 (PMC12732698; doi:10.3390/genes16121459)
Supplement: Supplementary file 1 [file genes-16-01459-s001.zip › genes-3970608-supplementary.pdf]

Supporting Information

Multi-Transcriptome-Informed Network Pharmacology Reveals Novel Biomarkers and Therapeutic Candidates for Parkinson's Disease

Md. Al Amin Pappu[1], Md. Alamin[2*], Md Al Noman[1,3], Most. Humaira Sultana[4], Md. Foysal Ahmed[1], Md. Sanoar Hossain[1], Md. Abdul Latif[1], Md. Fahim Faysal[5], AKM Azad[6,7], Salem A. Alyami[6,7], Naif Alotaibi[6,7], and Md. Nurul Haque Mollah[1*]

1 Bioinformatics Lab (Dry), Department of Statistics, University of Rajshahi, Rajshahi 6205, Bangladesh
2 Department of Mathematics and Physics, School of Engineering & Physical Sciences, North South University, Dhaka 1229, Bangladesh
3 Department of Statistics, University of Barishal, Barishal 8254, Bangladesh
4 Department of Management, School of Business & Economics, North South University, Dhaka 1229, Bangladesh
5 Department of Computer Science and Engineering (CSE), Rajshahi University of Engineering and Technology (RUET), Rajshahi 6203, Bangladesh
6 Department of Mathematics and Statistics, Faculty of Science, Imam Mohammad Ibn Saud Islamic University (IMSIU), Riyadh 13318, Saudi Arabia; kazad@imamu.edu.sa (A.A.)
7 Research Department, King Salman Center for Disability Research, Riyadh 11614, Saudi Arabia
* Correspondence: md.alamin06@northsouth.edu (M.A.); mollah.stat.bio@ru.ac.bd (M.N.H.M.)

Table of Contents
**Figure S1**. Pre- and Post-Integration Quality Control, MA plot and PCA visualization
**Figure S2.** Expression patterns of KGs with Boxplots for Parkinson's disease.
**Figure S3**. Gene Set Enrichment Analysis (GSEA) plots
**Table S1.** List of upregulated and downregulated DEGs in PD from 6 datasets (GSE8397-GPL96, GSE20141, GSE49036, GSE20163, GSE20292, and GSE20164)
**Table S2.** List of key genes (KGs) from PPI network based on different topological measures
**Table S3.** Verification of association between PD and KGs
**Table S4.** List of GO and KEGG terms based on Key Genes
**Table S5:** Collection of PD related candidate drug agents from published articles and others sources
**Table S6.** Docking/(binding affinity) scores (kcal/mol) between the proposed target genes/proteins (receptors) and top ordered 40 candidate drugs (out of 120).
**Table S7.** The 3-dimension view of strong binding interactions between targets and drugs
**References**

Supplementary Figure

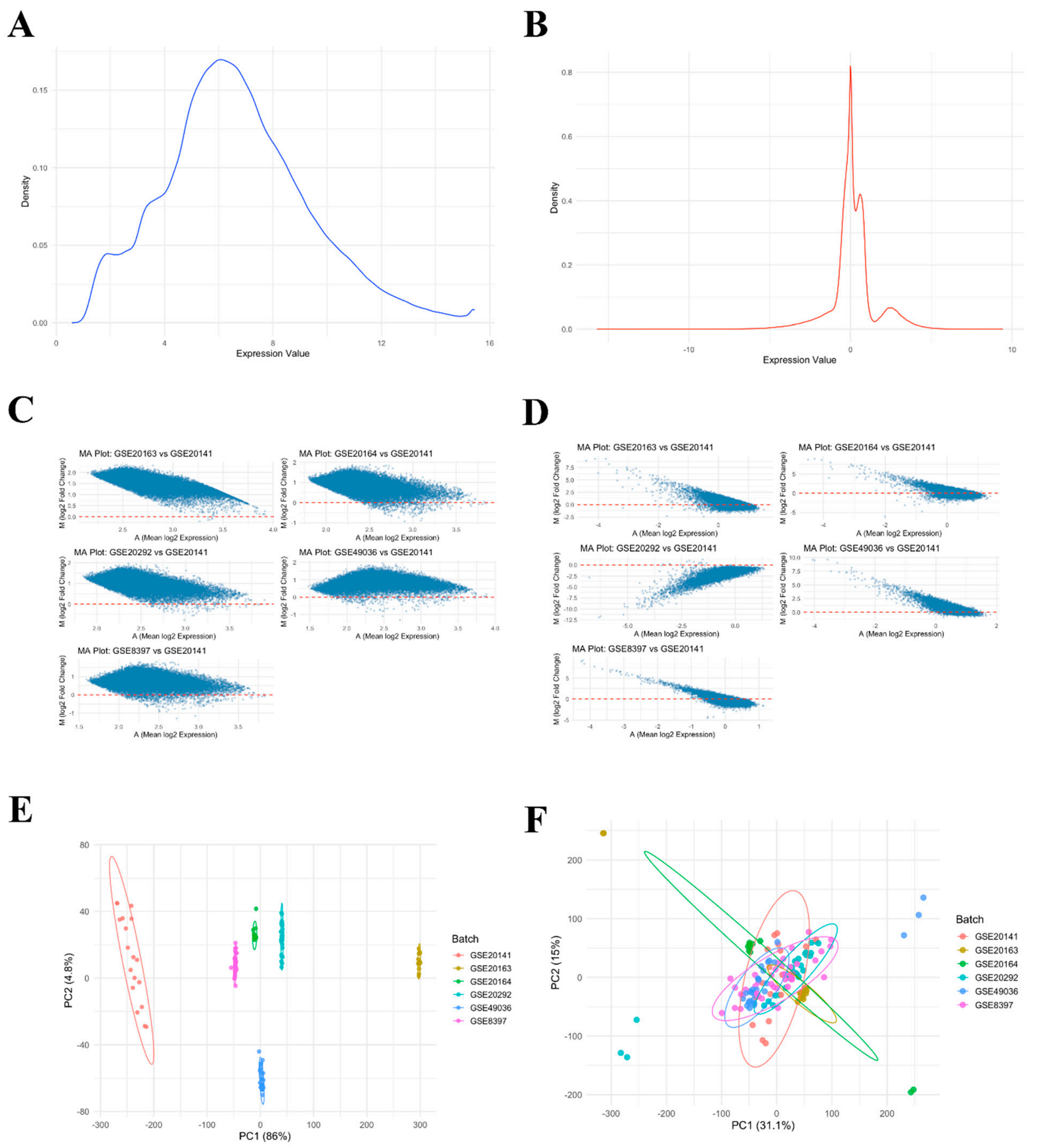

Figure S1. Quality Control and Batch-Effect Assessment Before and After Integration.

(A) Density plot of raw expression data showing skewed, non-uniform distributions. (B) Density plot after normalization showing improved, symmetric profiles. (C) MA-plot before normalization indicating intensity-dependent bias. (D) MA-plot after normalization showing removal of this bias. (E) PCA before batch correction illustrating clear clustering by dataset. (F) PCA after integration demonstrating reduced batch separation and improved mixing of samples. (G) Overview of the analysis pipeline, summarizing the major processing steps from raw data to downstream analyses.

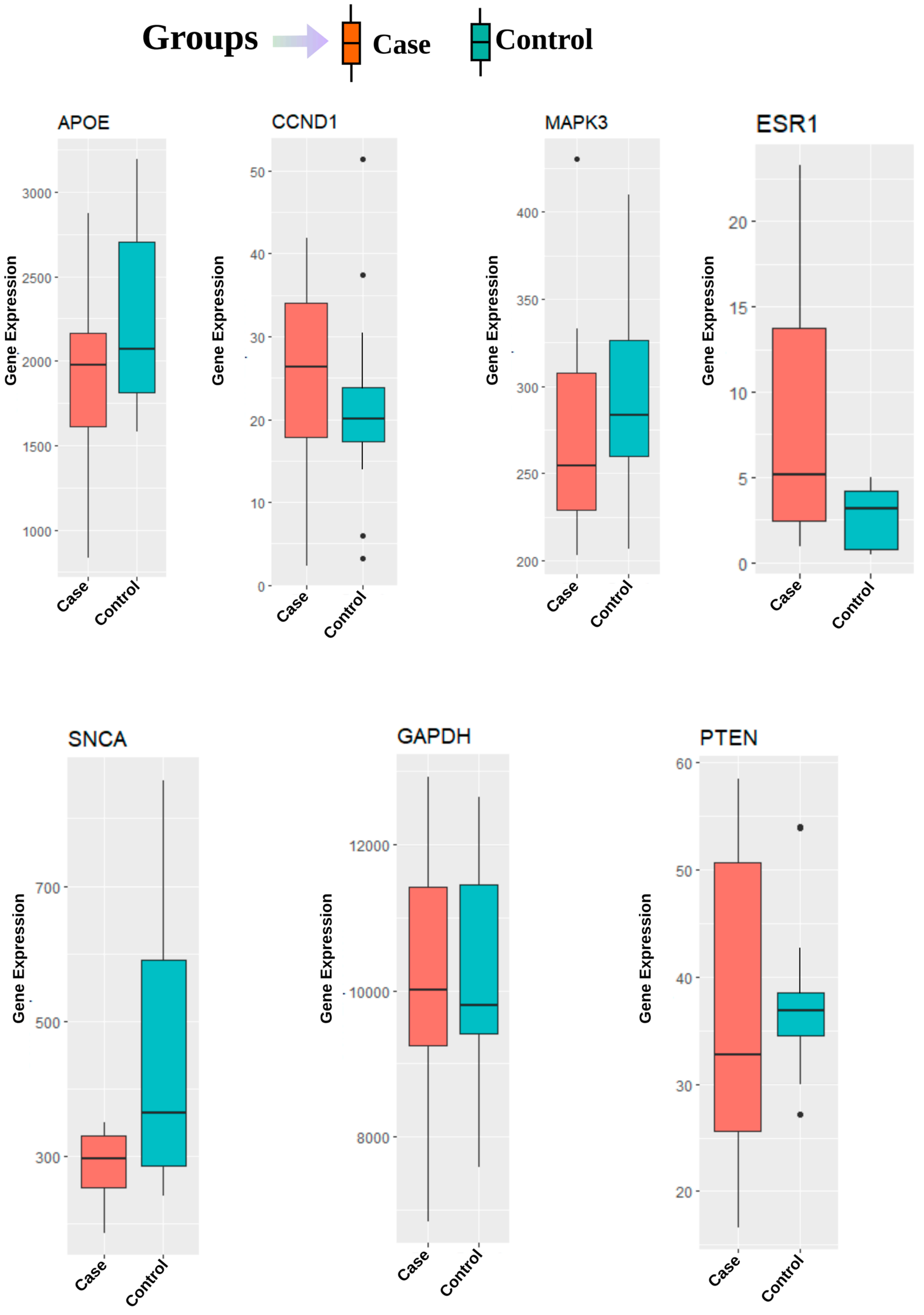

Figure S2. Expression patterns of KGs with Boxplots for Parkinson's disease (PD).

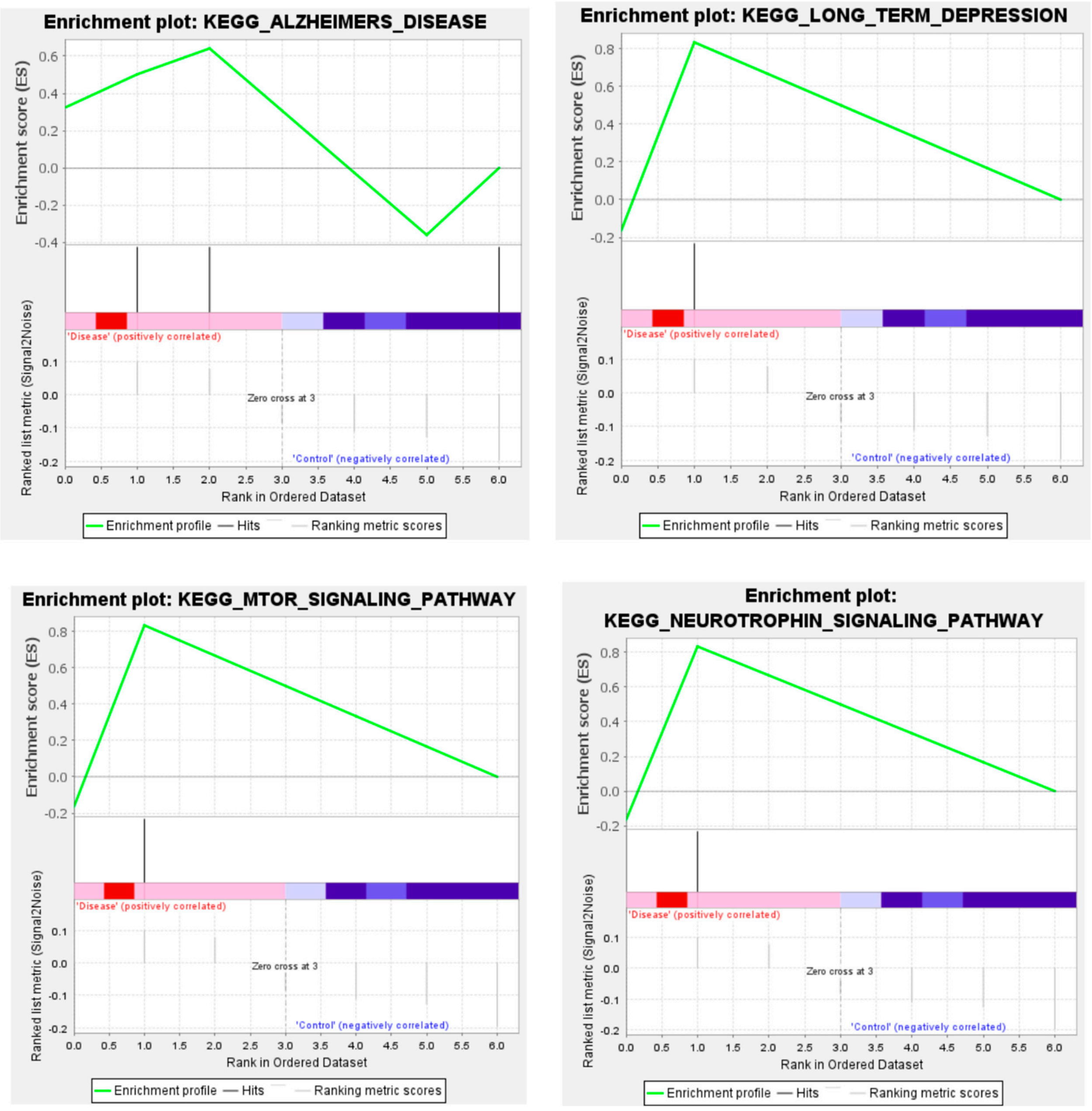

Figure S3. Gene Set Enrichment Analysis (GSEA) plots showing enrichment of KEGG pathways in the disease group compared to the control.

Supplementary Tables.

Table S1. List of upregulated and downregulated DEGs of PD from 6 datasets (GSE8397-GPL96, GSE20141, GSE49036, GSE20163, GSE20292, and GSE20164)

Upregulated DEGs	Downregulated DEGs
SH3PXD2A, LRP12, KLRA1P, SLC35C1, NGDN, PHC3, TSC2, AVPR2, KLF12, KRT86, ZNF747, MDM2, MOXD1, ZNF492, GLRX3, SLC6A6, CD69, UGT2B28, TPMT, TP53AIP1, ASPM, GYPE, HOXD3, SH2D3A, AKAP13, LHX2, SLC7A5P2, SPN, OVOL2, POLR2J4, PTPN11, OSR2, TOR4A, ZDHHC17, SINHCAF, BCL2L11, TARP, RBM14, DAPP1, VCPIP1, ESR1, TAF13, TNPO3, PRDX3, NSL1, CYB5A, CD36, STAM2, MSX2, EEF1D, MMP1, PPFIBP1, MYH9, ZNF675, AR, LPAR4, RAG1,	FTL, SDF4, ERGIC3, SNRPB, SRM, FXYD1, H2AX, WDR1, TNPO2, CCDC85B, ZNF91, CLPP, HYOU1, B2M, OAZ1, ARHGDIA, RPL3, GRINA, VAC14, ARL2, ATP1A2, UBC, NUCB1, MAPK3, KAT8, CST3, GPS2, PHB2, AIP, RPL18A, ABHD17A, DHPS, RAB31, MVP, SNRPA, MIF, MPG, MT1H, GSTP1, S100A6, FAM89B, CREB3, PLXNB2, SEC61A1, MAP2K2, HLA-B, LRP10, MT1HL1, RAD23A, SNCA, PRRC2A, TMEM222, RPL13A, ATP6V0C, BAG6, PPP1R11, GET3, PRDX2, BBLN, MGLL, ARFIP2, RPS10, CLEC16A, RPL13,

FABP2, OPHN1, SRSF5, SYN2, TNFSF9, GTF2I, PRO0255, LINC00328, POU4F1, RHD, ALDOAP2, CHD9, ZNF552, TFPI, POM121, EPB41, PWWP2A, SCAMP1, HCG9, RB1, MFAP3L, PRKCI, RECQL5, ZNF440, KRAS, MAP1A, TLE4, FSBP, FUBP1, KCNV1, MAN1A1, GTPBP3, GAPDH, TRIM23, UBE3A, NF1, VHL, DACH1, RCAN3, SNIP1, BMP5, ACVR1B, EEF1E1, MIS18A, FGF5, PDE4D, POU4F2, ZNF253, MCM4, DMC1, PEX13, ZNF157, SEC22B3P, COX5B, H3-3A, ADH1B, ARHGEF12, ARF6, GP6, ITGB5, INHBC, SPACA1, RMND5A, MAP2K3, SNRPE, SEL1L, TM2D1, FCAR, LIN28A, NDC80, WT1, CLCN4, DLG4, NUP62CL, EREG, RGPD8, EPS15L1, EPPIN, TWF1, GAGE12F, SCCPDH, EPB41L5, ZNF528, TRIM2, TFAP2C, HOXA10, GRM5, DAG1, PIWIL1, ERO1A, SMIM8, CCND1, SMG1P3, POLR2A, MLLT10, LINC01949, FN1, MAB21L2, KRT24, LSM4, C10orf95-AS1, RELN, STX6, METTL5, ID2B, CAMSAP1, ACAP2, SIKE1, GPRIN2, AQP3, CYP2C9, GABPB1, LRRC37BP1, SKIL, RIGI, CLCA2, CUL4B, ERAP1, SAP30, EIF5B, DNAH3, CYP2B6, MYLK3, ENPP1, KCNJ4, CSN1S1, KCNJ5, ADSS2, CPT1A, ATP6V0E1, APBB2, TPH1, FABP4, CELF1, SHOX2, SYCP1, ITSN1, PLXNC1, HIPK3, CDC14B, RO60, HNRNPH3, NPM1, IGF2BP3, SH3GL3, MCL1, TYMP	EIF3K, FKBP15, FLII, HGS, CIZ1, PC, NCSTN, PTGDS, MEN1, APOE, TRIM28, ADISSP, RPS2, STK11, NSMF, RHOC, RPS5, SORBS3, PINK1, MRPL4, NOL10, SNTA1, COPE, PNN, PTEN, SASH1, CAMK2G, DCXR, LMF1, PRAF2, KAT5, SERF2, RPL29, CNPY3, IRF2BP1

Table S2. List of key genes (KGs) from the PPI network based on different topological measures

SN	KGs	Betweenness	Closeness	Degree	EPC	MNC	Radiality	Stress
1	GAPDH	15229	155.03	72	50.98	69	8.09	76140
2	PTEN	2630.47	133.98	41	45.45	41	7.83	20056
3	CCND1	649.91	125.73	28	44.57	28	7.74	7342
4	APOE	3465.22	119.12	25	32.19	20	7.59	19412

5	ESR1	7516.19	136.45	48	45.26	42	7.83	41924
6	MAPK3	6071.79	135.12	42	45.57	36	7.85	31452
7	SNCA	1074.91	115.45	17	28.6	16	7.55	7490

Table S3. Verification of the association between PD and KGs

Terms	Overlap	P-value	Adjusted P-value	Genes
Astrocytoma	7/741	9.32E-11	1.98E-07	CCND1;PTEN;APOE;GAPDH;ESR1;MAPK3;SNCA
Thymoma	5/205	2.23E-09	2.36E-06	CCND1;PTEN;GAPDH;ESR1;MAPK3
Lymphoma	7/1307	5.01E-09	3.55E-06	CCND1;PTEN;APOE;GAPDH;ESR1;MAPK3;SNCA
Brain Neoplasms	6/646	7.56E-09	4.01E-06	CCND1;PTEN;APOE;GAPDH;ESR1;SNCA
Aortic Aneurysm, Abdominal	5/288	1.23E-08	4.34E-06	PTEN;APOE;GAPDH;ESR1;MAPK3
Anoxia	5/288	1.23E-08	4.34E-06	CCND1;APOE;GAPDH;ESR1;MAPK3
Central neuroblastoma	7/1655	2.63E-08	6.80E-06	CCND1;PTEN;APOE;GAPDH;ESR1;MAPK3;SNCA
Neuroblastoma	7/1698	3.14E-08	6.80E-06	CCND1;PTEN;APOE;GAPDH;ESR1;MAPK3;SNCA
Adenocarcinoma	7/1712	3.33E-08	6.80E-06	CCND1;PTEN;APOE;GAPDH;ESR1;MAPK3;SNCA
Carcinoma, Cribriform	4/115	3.58E-08	6.80E-06	CCND1;PTEN;APOE;ESR1
Carcinoma, Granular Cell	4/115	3.58E-08	6.80E-06	CCND1;PTEN;APOE;ESR1
Adenocarcinoma, Basal Cell	4/117	3.84E-08	6.80E-06	CCND1;PTEN;APOE;ESR1
Nephroblastoma	5/370	4.30E-08	6.90E-06	CCND1;PTEN;GAPDH;ESR1;MAPK3
Adenocarcinoma, Oxyphilic	4/122	4.55E-08	6.90E-06	CCND1;PTEN;APOE;ESR1
Rheumatoid Arthritis	7/1833	5.37E-08	7.14E-06	CCND1;PTEN;APOE;GAPDH;ESR1;MAPK3;SNCA
Neurilemmoma	4/135	6.84E-08	8.55E-06	CCND1;PTEN;MAPK3;SNCA
Alzheimer's Disease	7/82	9.30E-08	1.09E-05	CCND1;PTEN;APOE;GAPDH;ESR1;MAPK3;SNCA
Osteoporosis	5/441	1.03E-07	1.09E-05	CCND1;APOE;GAPDH;ESR1;MAPK3
Adenoma	6/1000	1.03E-07	1.09E-05	CCND1;PTEN;APOE;GAPDH;ESR1;MAPK3
Familial lichen amyloidosis	4/153	1.13E-07	1.09E-05	CCND1;PTEN;APOE;MAPK3
Squamous cell carcinoma of tongue	4/153	1.13E-07	1.09E-05	CCND1;PTEN;ESR1;MAPK3
Cholangiocarcinoma	5/455	1.21E-07	1.11E-05	CCND1;PTEN;APOE;ESR1;MAPK3
androgen independent prostate cancer	4/163	1.46E-07	1.27E-05	CCND1;PTEN;ESR1;MAPK3

Parkinson Disease	6/1064	1.49E-07	1.27E-05	PTEN;APOE;GAPDH;ESR1;MAPK3;SNCA
Colorectal Neoplasms	6/1073	1.57E-07	1.28E-05	CCND1;PTEN;APOE;GAPDH;ESR1;MAPK3
ACTH-Secreting Pituitary Adenoma	3/37	2.03E-07	1.58E-05	CCND1;GAPDH;MAPK3
Secondary malignant neoplasm of liver	5/508	2.09E-07	1.58E-05	CCND1;PTEN;GAPDH;ESR1;MAPK3
Hashimoto Disease	4/186	2.48E-07	1.72E-05	CCND1;PTEN;ESR1;MAPK3
Motor symptoms	3/40	2.58E-07	1.72E-05	PTEN;APOE;SNCA
Familial Alzheimer Disease (FAD)	4/188	2.59E-07	1.72E-05	CCND1;APOE;ESR1;SNCA

Table S4. List of GO and KEGG terms based on Key Genes.

Biological Process:

GO Terms	GO ID	P-value	Adjusted P-value	Associated Key Genes
Negative Regulation Of Wound Healing	GO:0061045	3.95E-05	0.005622	PTEN;APOE
Negative Regulation Of Epithelial Cell Migration	GO:0010633	5.52E-05	0.005622	PTEN;APOE
Regulation Of ERK1 And ERK2 Cascade	GO:0070372	6.05E-05	0.005622	PTEN;APOE;MAPK3
Negative Regulation Of Smooth Muscle Cell Proliferation	GO:0048662	6.95E-05	0.005622	PTEN;APOE
Negative Regulation Of Transport	GO:0051051	7.73E-05	0.005622	PTEN;SNCA
Regulation Of Protein Phosphorylation	GO:0001932	7.74E-05	0.005622	CCND1;PTEN;MAPK3
Negative Regulation Of Secretion By Cell	GO:1903531	8.14E-05	0.005622	APOE;SNCA
Chemical Synaptic Transmission	GO:0007268	8.45E-05	0.005622	PTEN;APOE;SNCA
Positive Regulation Of Transport	GO:0051050	1.28E-04	0.006788	APOE;SNCA
Negative Regulation Of Cell Projection Organization	GO:0031345	1.28E-04	0.006788	PTEN;APOE
Negative Regulation Of Neuron Projection Development	GO:0010977	1.49E-04	0.006936	PTEN;APOE
Negative Regulation Of Gene Expression	GO:0010629	1.56E-04	0.006936	APOE;ESR1;GAPDH
Positive Regulation Of Protein Phosphorylation	GO:0001934	2.20E-04	0.007903	CCND1;MAPK3;SNCA
Regulation Of Endocytosis	GO:0030100	2.23E-04	0.007903	APOE;SNCA
Response To Reactive Oxygen Species	GO:0000302	2.23E-04	0.007903	APOE;MAPK3
Negative Regulation Of Supramolecular Fiber Organization	GO:1902904	2.58E-04	0.008577	APOE;SNCA
Regulation Of Cyclin-Dependent Protein Serine/Threonine Kinase Activity	GO:0000079	3.19E-04	0.008712	CCND1;PTEN
Positive Regulation Of Endocytosis	GO:0045807	3.19E-04	0.008712	APOE;SNCA
Negative Regulation Of Protein	GO:0071901	3.28E-04	0.008712	PTEN;APOE

Serine/Threonine Kinase Activity				
Response To Type II Interferon	GO:0034341	3.28E-04	0.008712	GAPDH;SNCA
Negative Regulation Of Neuron Death	GO:1901215	3.96E-04	0.010038	APOE;SNCA
Regulation Of Peptidyl-Serine Phosphorylation	GO:0033135	4.42E-04	0.010234	PTEN;SNCA
Negative Regulation Of Amide Metabolic Process	GO:0034249	4.42E-04	0.010234	APOE;GAPDH
Negative Regulation Of MAPK Cascade	GO:0043409	5.64E-04	0.012492	PTEN;APOE
Response To Lipid	GO:0033993	6.18E-04	0.012823	ESR1;SNCA
Regulation Of Protein Serine/Threonine Kinase Activity	GO:0071900	6.41E-04	0.012823	CCND1;SNCA
Positive Regulation Of Cellular Component Organization	GO:0051130	6.52E-04	0.012823	APOE;SNCA
Positive Regulation Of Protein Serine/Threonine Kinase Activity	GO:0071902	6.99E-04	0.012823	CCND1;SNCA
Cellular Response To Oxidative Stress	GO:0034599	6.99E-04	0.012823	MAPK3;SNCA
Receptor-Mediated Endocytosis	GO:0006898	7.60E-04	0.013471	APOE;SNCA
Synapse Organization	GO:0050808	8.75E-04	0.015017	PTEN;SNCA
Cellular Response To Metal Ion	GO:0071248	9.29E-04	0.015443	MAPK3;SNCA
Positive Regulation Of Transferase Activity	GO:0051347	0.001041	0.016243	APOE;MAPK3
Negative Regulation Of Response To External Stimulus	GO:0032102	0.001085	0.016243	PTEN;APOE
Positive Regulation Of Cellular Catabolic Process	GO:0031331	0.001085	0.016243	APOE;SNCA
Negative Regulation Of Protein Metabolic Process	GO:0051248	0.0011	0.016243	APOE;GAPDH
Negative Regulation Of Protein Phosphorylation	GO:0001933	0.00113	0.016243	PTEN;SNCA
Regulation Of Neuron Projection Development	GO:0010975	0.001536	0.016493	PTEN;APOE
Positive Regulation Of ERK1 And ERK2 Cascade	GO:0070374	0.001624	0.016493	APOE;MAPK3
Negative Regulation Of Transcription By RNA Polymerase II	GO:0000122	0.001725	0.016493	CCND1;ESR1;SNCA
Negative Regulation Of Excitatory Postsynaptic Potential	GO:0090394	0.001749	0.016493	PTEN
Regulation Of Macrophage Proliferation	GO:0120040	0.001749	0.016493	MAPK3
Positive Regulation Of Vesicle Fusion	GO:0031340	0.001749	0.016493	SNCA
Postsynaptic Density Assembly	GO:0097107	0.001749	0.016493	PTEN
Lipoprotein Metabolic Process	GO:0042157	0.001749	0.016493	APOE
Prepulse Inhibition	GO:0060134	0.001749	0.016493	PTEN
Negative Regulation Of Cell Size	GO:0045792	0.001749	0.016493	PTEN
Positive Regulation Of Extrinsic Apoptotic Signaling Pathway Via Death Domain Receptors	GO:1902043	0.001749	0.016493	PTEN
Negative Regulation Of Macromolecule Metabolic Process	GO:0010605	0.001752	0.016493	APOE;ESR1
Endocytosis	GO:0006897	0.001808	0.016493	APOE;MAPK3

Neuron Projection Development	GO:0031175	0.001865	0.016493	PTEN;APOE
Negative Regulation Of Dendritic Spine Development	GO:0061000	0.002098	0.016493	PTEN
Sterol Catabolic Process	GO:0016127	0.002098	0.016493	APOE
Synaptic Transmission, Dopaminergic	GO:0001963	0.002098	0.016493	SNCA
Regulation Of Inositol Phosphate Biosynthetic Process	GO:0010919	0.002098	0.016493	SNCA
Positive Regulation Of Receptor Catabolic Process	GO:2000646	0.002098	0.016493	APOE
Positive Regulation Of Steroid Metabolic Process	GO:0045940	0.002098	0.016493	APOE
Locomotor Rhythm	GO:0045475	0.002098	0.016493	PTEN
Cholesterol Catabolic Process	GO:0006707	0.002098	0.016493	APOE
Positive Regulation Of Amyloid-Beta Clearance	GO:1900223	0.002098	0.016493	APOE
Protein Stabilization	GO:0050821	0.002184	0.016493	PTEN;GAPDH
Regulation Of Chaperone-Mediated Autophagy	GO:1904714	0.002448	0.016493	SNCA
Regulation Of Dopamine Uptake Involved In Synaptic Transmission	GO:0051584	0.002448	0.016493	SNCA
Regulation Of Extent Of Cell Growth	GO:0061387	0.002448	0.016493	APOE
Negative Regulation Of Monooxygenase Activity	GO:0032769	0.002448	0.016493	SNCA
Very-Low-Density Lipoprotein Particle Remodeling	GO:0034372	0.002448	0.016493	APOE
Regulation Of Lipase Activity	GO:0060191	0.002448	0.016493	SNCA
Postsynaptic Density Organization	GO:0097106	0.002448	0.016493	PTEN
Cellular Response To Epinephrine Stimulus	GO:0071872	0.002448	0.016493	SNCA
Positive Regulation Of CoA-transferase Activity	GO:1905920	0.002448	0.016493	APOE
Regulation Of Cdc42 Protein Signal Transduction	GO:0032489	0.002448	0.016493	APOE
Regulation Of CoA-transferase Activity	GO:1905918	0.002448	0.016493	APOE
Regulation Of Wound Healing, Spreading Of Epidermal Cells	GO:1903689	0.002448	0.016493	PTEN
Response To Epinephrine	GO:0071871	0.002448	0.016493	SNCA
Dopamine Uptake	GO:0090494	0.002448	0.016493	SNCA
Positive Regulation Of Protein Modification Process	GO:0031401	0.002549	0.016493	CCND1;MAPK3
Negative Regulation Of Intracellular Signal Transduction	GO:1902532	0.002685	0.016493	PTEN;ESR1
Positive Regulation Of Phosphorylation	GO:0042327	0.002685	0.016493	CCND1;MAPK3
Regulation Of I-kappaB kinase/NF-kappaB Signaling	GO:0043122	0.002708	0.016493	ESR1;GAPDH
Positive Regulation Of Inositol Phosphate Biosynthetic Process	GO:0060732	0.002797	0.016493	SNCA
Negative Regulation Of Long-Term Synaptic Potentiation	GO:1900272	0.002797	0.016493	APOE
Triglyceride-Rich Lipoprotein Particle Remodeling	GO:0034370	0.002797	0.016493	APOE

Alcohol Catabolic Process	GO:0046164	0.002797	0.016493	APOE
Negative Regulation Of Transporter Activity	GO:0032410	0.002797	0.016493	SNCA
Positive Regulation Of Ubiquitin Protein Ligase Activity	GO:1904668	0.002797	0.016493	PTEN
Regulation Of Organ Growth	GO:0046620	0.002797	0.016493	PTEN
Presynaptic Membrane Assembly	GO:0097105	0.002797	0.016493	PTEN

Cellular Component:

GO Terms	GO ID	P-value	Adjusted P-value	Associated Key Genes
Nucleus	GO:0005634	2.85E-05	0.00134	CCND1;PTEN;APOE;ESR1;GAPDH;MAPK3;SNCA
Intracellular Membrane-Bounded Organelle	GO:0043231	7.74E-05	0.001819	CCND1;PTEN;APOE;ESR1;GAPDH;MAPK3;SNCA
Neuron Projection	GO:0043005	6.92E-04	0.010046	PTEN;APOE;SNCA
Cytoskeleton	GO:0005856	8.55E-04	0.010046	GAPDH;MAPK3;SNCA
Low-Density Lipoprotein Particle	GO:0034362	0.002098	0.01878	APOE
Vesicle	GO:0031982	0.002777	0.01878	APOE;GAPDH
Chylomicron	GO:0042627	0.002797	0.01878	APOE
Endoplasmic Reticulum Lumen	GO:0005788	0.004025	0.020895	APOE;MAPK3
Early Endosome	GO:0005769	0.004687	0.020895	APOE;MAPK3
Triglyceride-Rich Plasma Lipoprotein Particle	GO:0034385	0.00489	0.020895	APOE
Very-Low-Density Lipoprotein Particle	GO:0034361	0.00489	0.020895	APOE
Platelet Alpha Granule Membrane	GO:0031092	0.005587	0.021884	SNCA
High-Density Lipoprotein Particle	GO:0034364	0.006284	0.022719	APOE
Endocytic Vesicle Lumen	GO:0071682	0.007328	0.024601	APOE
Melanosome	GO:0042470	0.010801	0.033844	APOE
Supramolecular Fiber	GO:0099512	0.012534	0.036818	SNCA
Cyclin-Dependent Protein Kinase Holoenzyme Complex	GO:0000307	0.013918	0.03848	CCND1
Euchromatin	GO:0000791	0.015301	0.039952	ESR1
Multivesicular Body	GO:0005771	0.017372	0.042972	APOE
Extracellular Membrane-Bounded Organelle	GO:0065010	0.019783	0.043939	APOE
Serine/Threonine Protein Kinase Complex	GO:1902554	0.020815	0.043939	CCND1
Extracellular Vesicle	GO:1903561	0.020815	0.043939	APOE
Caveola	GO:0005901	0.021502	0.043939	MAPK3
Clathrin-Coated Endocytic Vesicle Membrane	GO:0030669	0.023562	0.046142	APOE
Glutamatergic Synapse	GO:0098978	0.026986	0.048696	APOE
Lipid Droplet	GO:0005811	0.028353	0.048696	GAPDH
Plasma Membrane Raft	GO:0044853	0.028353	0.048696	MAPK3
Clathrin-Coated Endocytic Vesicle	GO:0045334	0.029378	0.048696	APOE
Platelet Alpha Granule	GO:0031091	0.030742	0.048696	SNCA
Clathrin-Coated Vesicle Membrane	GO:0030665	0.031082	0.048696	APOE
Intracellular Organelle Lumen	GO:0070013	0.033294	0.050478	APOE;MAPK3
Cytoplasmic Side Of Plasma Membrane	GO:0009898	0.042607	0.062579	PTEN

Molecular Function:

GO Terms	GO ID	P-value	Adjusted P-value	Associated Key Genes
Tau Protein Binding	GO:0048156	7.73E-05	0.005413	APOE;SNCA

Protein Kinase Regulator Activity	GO:0019887	5.42E-04	0.018983	CCND1;SNCA
Telomerase Activity	GO:0003720	0.001749	0.020699	PTEN
Phosphatidylcholine-Sterol O-acyltransferase Activator Activity	GO:0060228	0.001749	0.020699	APOE
RNA-directed DNA Polymerase Activity	GO:0003964	0.001749	0.020699	PTEN
Phosphatidylinositol Trisphosphate Phosphatase Activity	GO:0034594	0.002098	0.020699	PTEN
Aspartic-Type Endopeptidase Inhibitor Activity	GO:0019828	0.002098	0.020699	GAPDH
Cuprous Ion Binding	GO:1903136	0.002797	0.020699	SNCA
RNA Polymerase II General Transcription Initiation Factor Binding	GO:0001091	0.003146	0.020699	ESR1
Phospholipase Inhibitor Activity	GO:0004859	0.003146	0.020699	SNCA
Estrogen Response Element Binding	GO:0034056	0.003495	0.020699	ESR1
MAP Kinase Activity	GO:0004707	0.003844	0.020699	MAPK3
Phosphatidylinositol-3-Phosphate Phosphatase Activity	GO:0004438	0.003844	0.020699	PTEN
Phosphatidylinositol Monophosphate Phosphatase Activity	GO:0052744	0.004193	0.020965	PTEN
Cyclin-Dependent Protein Serine/Threonine Kinase Activator Activity	GO:0061575	0.00489	0.022505	CCND1
Tubulin Binding	GO:0015631	0.005144	0.022505	GAPDH;SNCA
TBP-class Protein Binding	GO:0017025	0.006284	0.025529	ESR1
Cysteine-Type Endopeptidase Inhibitor Activity Involved In Apoptotic Process	GO:0043027	0.006632	0.025529	SNCA
Ubiquitin-Specific Protease Binding	GO:1990381	0.00698	0.025529	PTEN
Telomerase RNA Binding	GO:0070034	0.007328	0.025529	PTEN
Low-Density Lipoprotein Particle Receptor Binding	GO:0050750	0.007676	0.025529	APOE
Disordered Domain Specific Binding	GO:0097718	0.008023	0.025529	GAPDH
Ferrous Iron Binding	GO:0008198	0.008718	0.026534	SNCA
Lipoprotein Particle Receptor Binding	GO:0070325	0.009413	0.026738	APOE
Kinase Binding	GO:0019900	0.010268	0.026738	CCND1;ESR1
General Transcription Initiation Factor Binding	GO:0140296	0.010454	0.026738	ESR1
Kinesin Binding	GO:0019894	0.010801	0.026738	SNCA
Transcription Coactivator Binding	GO:0001223	0.011841	0.026738	ESR1
Nuclear Estrogen Receptor Binding	GO:0030331	0.011841	0.026738	ESR1
Hsp70 Protein Binding	GO:0030544	0.011841	0.026738	SNCA
Kinase Inhibitor Activity	GO:0019210	0.011841	0.026738	SNCA
Protein Kinase Binding	GO:0019901	0.012565	0.027231	CCND1;ESR1
Metal Ion Binding	GO:0046872	0.01304	0.027231	APOE;SNCA
Alpha-Tubulin Binding	GO:0043014	0.013226	0.027231	SNCA
Transcription Corepressor Binding	GO:0001222	0.01461	0.02922	ESR1
Protein Kinase Inhibitor Activity	GO:0004860	0.015646	0.029458	SNCA
Copper Ion Binding	GO:0005507	0.015646	0.029458	SNCA
Cyclin-Dependent Protein Serine/Threonine Kinase Regulator Activity	GO:0016538	0.015992	0.029458	CCND1
Protein Serine/Threonine Phosphatase Activity	GO:0004722	0.019095	0.033188	PTEN
Protein Serine/Threonine Kinase Activator Activity	GO:0043539	0.019439	0.033188	CCND1
Iron Ion Binding	GO:0005506	0.019439	0.033188	SNCA
PDZ Domain Binding	GO:0030165	0.021502	0.035837	PTEN
Sequence-Specific Double-Stranded DNA Binding	GO:1990837	0.023783	0.038716	ESR1;SNCA
ATPase Binding	GO:0051117	0.025276	0.040211	ESR1
Protein Tyrosine Phosphatase Activity	GO:0004725	0.026302	0.040915	PTEN
Amyloid-Beta Binding	GO:0001540	0.027328	0.041587	APOE

Transcription Coregulator Binding	GO:0001221	0.034145	0.049794	ESR1
Histone Deacetylase Binding	GO:0042826	0.034145	0.049794	CCND1
Phosphatase Binding	GO:0019902	0.038553	0.053974	MAPK3
Endopeptidase Inhibitor Activity	GO:0004866	0.038553	0.053974	GAPDH
Nuclear Receptor Binding	GO:0016922	0.039568	0.054309	ESR1
Protease Binding	GO:0002020	0.042944	0.05781	PTEN

KEGG Pathways:

Terms	P-value	Adjusted P-value	Associated Key Genes
Breast cancer	9.64E-08	1.28E-05	CCND1;PTEN;ESR1;MAPK3
Endometrial cancer	8.03E-07	4.66E-05	CCND1;PTEN;MAPK3
Prolactin signaling pathway	1.42E-06	4.66E-05	CCND1;ESR1;MAPK3
Melanoma	1.55E-06	4.66E-05	CCND1;PTEN;MAPK3
Glioma	1.75E-06	4.66E-05	CCND1;PTEN;MAPK3
Prostate cancer	3.82E-06	7.26E-05	CCND1;PTEN;MAPK3
Alzheimer disease	3.82E-06	7.26E-05	APOE;GAPDH;MAPK3;SNCA
Thyroid hormone signaling pathway	7.43E-06	1.23E-04	CCND1;ESR1;MAPK3
FoxO signaling pathway	9.43E-06	1.39E-04	CCND1;PTEN;MAPK3
Cellular senescence	1.59E-05	1.95E-04	CCND1;PTEN;MAPK3
Pathways in cancer	1.61E-05	1.95E-04	CCND1;PTEN;ESR1;MAPK3
Hepatocellular carcinoma	1.99E-05	2.20E-04	CCND1;PTEN;MAPK3
Focal adhesion	3.40E-05	3.42E-04	CCND1;PTEN;MAPK3
Proteoglycans in cancer	3.60E-05	3.42E-04	CCND1;ESR1;MAPK3
Human T-cell leukemia virus 1 infection	4.39E-05	3.89E-04	CCND1;PTEN;MAPK3
Chemical carcinogenesis	5.69E-05	4.73E-04	CCND1;ESR1;MAPK3
Thyroid cancer	6.95E-05	5.44E-04	CCND1;MAPK3
Bladder cancer	8.55E-05	6.32E-04	CCND1;MAPK3
MicroRNAs in cancer	1.23E-04	8.63E-04	CCND1;PTEN;MAPK3
Human papillomavirus infection	1.50E-04	9.95E-04	CCND1;PTEN;MAPK3
PI3K-Akt signaling pathway	1.83E-04	0.001156	CCND1;PTEN;MAPK3
Acute myeloid leukemia	2.30E-04	0.001388	CCND1;MAPK3
Central carbon metabolism in cancer	2.51E-04	0.00145	PTEN;MAPK3
Non-small cell lung cancer	2.65E-04	0.001451	CCND1;MAPK3
p53 signaling pathway	2.73E-04	0.001451	CCND1;PTEN
Chronic myeloid leukemia	2.96E-04	0.001456	CCND1;MAPK3
Pancreatic cancer	2.96E-04	0.001456	CCND1;MAPK3
Colorectal cancer	3.78E-04	0.001798	CCND1;MAPK3
PD-L1 expression and PD-1 checkpoint pathway in cancer	4.05E-04	0.001859	PTEN;MAPK3
Small cell lung cancer	4.33E-04	0.00192	CCND1;PTEN
AGE-RAGE signaling pathway in diabetic complications	5.11E-04	0.002194	CCND1;MAPK3
HIF-1 signaling pathway	6.07E-04	0.002523	GAPDH;MAPK3
Sphingolipid signaling pathway	7.23E-04	0.002914	PTEN;MAPK3
Apelin signaling pathway	9.56E-04	0.003533	CCND1;MAPK3
Autophagy	9.56E-04	0.003533	PTEN;MAPK3
Estrogen signaling pathway	9.56E-04	0.003533	ESR1;MAPK3
Gastric cancer	0.00113	0.004061	CCND1;MAPK3
mTOR signaling pathway	0.001206	0.004062	PTEN;MAPK3
Oxytocin signaling pathway	0.001206	0.004062	CCND1;MAPK3
Cushing syndrome	0.001222	0.004062	CCND1;MAPK3
Hepatitis C	0.001253	0.004065	CCND1;MAPK3
Kaposi sarcoma-associated herpesvirus	0.001884	0.005967	CCND1;MAPK3

infection			
Pathogenic Escherichia coli infection	0.001962	0.006069	GAPDH;MAPK3
Viral carcinogenesis	0.002082	0.006153	CCND1;MAPK3
Diabetic cardiomyopathy	0.002082	0.006153	PTEN;GAPDH
Human cytomegalovirus infection	0.002549	0.007371	CCND1;MAPK3
Salmonella infection	0.003111	0.008804	GAPDH;MAPK3
Pathways of neurodegeneration	0.010921	0.030261	MAPK3;SNCA
Aldosterone-regulated sodium reabsorption	0.01288	0.03496	MAPK3
Type II diabetes mellitus	0.015992	0.042538	MAPK3
Cholesterol metabolism	0.017372	0.045303	APOE
Endocrine and other factor-regulated calcium reabsorption	0.018406	0.047076	ESR1
Hedgehog signaling pathway	0.019439	0.048781	CCND1
VEGF signaling pathway	0.020471	0.049435	MAPK3
Viral myocarditis	0.020815	0.049435	CCND1
Long-term depression	0.020815	0.049435	MAPK3
GnRH secretion	0.022189	0.051775	MAPK3
Glycolysis / Gluconeogenesis	0.023219	0.05212	GAPDH
Long-term potentiation	0.023219	0.05212	MAPK3
Fc epsilon RI signaling pathway	0.023562	0.05212	MAPK3
Renal cell carcinoma	0.023905	0.05212	MAPK3
Adherens junction	0.02459	0.05275	MAPK3
Inositol phosphate metabolism	0.025276	0.053359	PTEN
Pertussis	0.026302	0.054519	MAPK3
Leishmaniasis	0.026644	0.054519	MAPK3
B cell receptor signaling pathway	0.028012	0.056448	MAPK3
ErbB signaling pathway	0.029378	0.057973	MAPK3
Gap junction	0.030401	0.057973	MAPK3
Th1 and Th2 cell differentiation	0.031764	0.057973	MAPK3
GnRH signaling pathway	0.032104	0.057973	MAPK3
TGF-beta signaling pathway	0.032444	0.057973	MAPK3
IL-17 signaling pathway	0.032444	0.057973	MAPK3
Circadian entrainment	0.033465	0.057973	MAPK3
Fc gamma R-mediated phagocytosis	0.033465	0.057973	MAPK3
Phosphatidylinositol signaling system	0.033465	0.057973	PTEN
Choline metabolism in cancer	0.033805	0.057973	MAPK3
Progesterone-mediated oocyte maturation	0.034484	0.057973	MAPK3
Melanogenesis	0.034824	0.057973	MAPK3
Chagas disease	0.035163	0.057973	MAPK3
T cell receptor signaling pathway	0.035842	0.057973	MAPK3
Toll-like receptor signaling pathway	0.035842	0.057973	MAPK3
C-type lectin receptor signaling pathway	0.035842	0.057973	MAPK3
Parathyroid hormone synthesis, secretion and action	0.03652	0.057973	MAPK3
Th17 cell differentiation	0.036859	0.057973	MAPK3
Insulin resistance	0.037198	0.057973	PTEN
TNF signaling pathway	0.038553	0.057973	MAPK3
Toxoplasmosis	0.038553	0.057973	MAPK3
Serotonergic synapse	0.038891	0.057973	MAPK3
Cholinergic synapse	0.038891	0.057973	MAPK3
Glutamatergic synapse	0.03923	0.057973	MAPK3
Growth hormone synthesis, secretion and action	0.04092	0.059002	MAPK3

Neurotrophin signaling pathway	0.04092	0.059002	MAPK3
AMPK signaling pathway	0.041257	0.059002	CCND1
Cell cycle	0.042607	0.05965	CCND1
Platelet activation	0.042607	0.05965	MAPK3
Osteoclast differentiation	0.043618	0.060111	MAPK3
Oocyte meiosis	0.044292	0.060111	MAPK3
Relaxin signaling pathway	0.044292	0.060111	MAPK3

Table S5. Collection of PD-related candidate drug agents from published articles and other sources

Paper title with reference	Drug List
Emerging drugs for Parkinson's disease [1]	Amantadine, Apomorphine, Benzatropine, Bromocriptine, Carbidopa, Entacapone, Pergolide, Pramipexole, Procyclidine, Ropinirole, Selegiline, Tolcapone, Trihexyphenidyl, levodopa
Medications for Parkinson's Disease [2]	Pimavanserin, rotigotine, rasagiline, Safinamide, opicapone,
Amantadine in the Treatment of Parkinson's Disease [3]	Amantadine
Identification of potential natural neuroprotective molecules for Parkinson's disease by using chemoinformatics and molecular docking [4]	Amphetamine, Cocaine, Methamphetamine, Rotenone, Baicalein, Caffeine, Curcumin, Epigallocatechin gallate, Metformin, Myricetin, Nordihydroguaiaretic acid, Nortriptyline, Scutellarein
Anti-Parkinson's disease drugs and pharmacogenetic considerations [5]	Benztropine, Biperiden, Diphenhydramine, Ethopropazine, Orphenadrine, Procyclidine, Trihexyphenidyl, Levodopa, Carbidopa, Benserazide, Bromocriptine, Lisuride, Pergolide, Cabergoline, Ropinirole, Pramipexole, Rotigotine, Apomorphine, Selegiline, Rasagiline, Entacapone, Tolcapone
NOVEL DRUGS FOR PARKINSON'S DISEASE [6]	Apomorphine, SIB-1508Y, OPC-14117, L-dopa
Muscarinic receptor binding changes in postmortem Parkinson's disease [7]	pirenzepine, 4DAMP, and, AF-DX 384
DSigDB: drug signatures database for gene set analysis [8]	Tanespimycin, disulfiram, alvespimycin, monorden, securinine, 1,4-chrysene-quinone, Elesclomol, tanespimycin, geldanamycin, raloxifene, Radicicol, thimerosal, thiostrepton, cycloheximide, cadmium sulfate, zinc acetate dihydrate, lomustine, parthenolide, aluminium sulfate, minocycline, crotonaldehyde, puromycin, LY-294002, MG-132, valproic acid, etacrynic acid, levonorgestrel, MG-262, N-acetyl-L-cysteine, albendazole, mometasone, 2-methylcholine, Mehp, spiperone, withaferin A, mesalazine, sanguinarine, gefitinib, ellipticine, trichostatin A, vorinostat, clotrimazole, celastrol, beta-carotene, metoprolol, azacitidine, irinotecan, fisetin, GW-8510, mebendazole, phenoxybenzamine, benzene, staurosporine, scriptaid, latrepirdine, 1,9-pyrazoloanthrone, meclofenoxate, 8-azaguanine, flunixin, mirtazapine, emetine, bortezomib, cyclophosphamide, propanil, pyrene, anisomycin, ABT-737, ginsenoside Re, cyclosporin A, doxorubicin, thapsigargin, ezetimibe, remifentanil, sirtinol
Drug repurposing in Parkinson's disease [9]	Ambroxol, Isradipine, Inosine, Ursodeoxycholic Acid (UDCA), Deferiprone, Exenatide, Nilotinib, Simvastatin
DGIdb: mining the druggable genome [10]	DONEPEZIL, GALANTAMINE, NICOTINE POLACRILEX, GANCICLOVIR, BUPROPION HYDROCHLORIDE, RIVASTIGMINE, PREDNISONE, GINKGO BILOBA EXTRACT, SOYBEAN, RITONAVIR, LORAZEPAM, MYCOPHENOLATE,

	KETOCONAZOLE, ACETAZOLAMIDE, TAMOXIFEN, HISTAMINE, ETHOXZOLAMIDE, CHLORTHALIDONE, ZONISAMIDE, METHOCARBAMOL, METHAZOLAMIDE, SULPIRIDE, DEFEROXAMINE, HISTIDINE, DORZOLAMIDE, BRINZOLAMIDE, PRASTERONE, SORAFENIB, ETOPOSIDE, SELENIUM, QUERCETIN, NITROGLYCERIN, ACETAMINOPHEN, SELADELPAR, TROGLITAZONE, NIFEDIPINE, ROSIGLITAZONE, FLUOROURACIL, RESVERATROL, BORTEZOMIB, MESALAMINE, ZAFIRLUKAST, CYTARABINE, LAPATINIB, CISPLATIN

Table S6. Docking scores (binding affinities, kcal/mol) between the proposed receptors and top-ordered 40 candidate drugs (out of 120)

Drugs	MAPK3	PTEN	GAPDH	TFAP2A	CCND1	APOE	FOXC1	SNCA	ESR1	NFKB 1
Nilotinib	-12.1	-9.6	-10	-8.7	-8.2	-8.5	-7.3	-7.1	-7.1	-6.5
Bromocriptine	-10.9	-8.7	-9.4	-8.3	-8.3	-7.7	-7.7	-7.1	-6.3	-6.2
withaferin A	-10	-10	-9	-8.7	-7.1	-7.2	-7.4	-7.2	-5.8	-6.4
celastrol	-9.7	-10.2	-8.4	-8.5	-8.1	-7.2	-7.6	-7.1	-5.2	-5.6
Donepezil	-9.7	-8.7	-8.2	-7.5	-7.3	-7.1	-7.1	-7.1	-6.2	-5
GW 8510	-10	-8.8	-9	-7.9	-7.5	-7.7	-7.4	-6.5	-6.1	-5.5
OPC 14117	-10.2	-8.9	-8.7	-8	-7.6	-7.6	-7	-5.4	-5.8	-6
ABT 737	-9.8	-9.6	-10.1	-8	-6	-8.2	-7.4	-6	-4.5	-4.8
Qinestrol	-8.8	-9.4	-8.1	-7.9	-7.5	-6.9	-6.9	-6.4	-6.9	-5.4
Enzalutamide	-9.5	-9	-8.4	-7.4	-7.6	-7.9	-6.8	-6.5	-5.5	-5.6
Gedunin	-9.3	-8.8	-8.7	-8.3	-7.7	-6.9	-7.4	-6.5	-5.1	-5.2
Staurosporine	-9.6	-8.9	-8.5	-8	-7.4	-7.2	-7.2	-6.7	-5.1	-5.3
Alpelisib	-9.5	-8.8	-8.5	-8	-7.9	-7.2	-6.7	-6.1	-5.9	-5
Estriol	-9.9	-7.9	-8.1	-7.1	-7.7	-7	-6.9	-6.6	-5.7	-5
Cortisol Acetate	-9.2	-9	-8.1	-7.4	-7.3	-6.2	-6.9	-6.7	-6	-5.1
Estropipate	-10.2	-8.6	-7.8	-7.4	-7	-6.4	-7.1	-5.9	-5.8	-5.2
Ketoconazole	-8.9	-8.7	-8.5	-7.8	-7.2	-7	-6.6	-5.7	-5.6	-5.4
Irbesartan	-8.8	-8.4	-8.3	-7.5	-7.8	-6.7	-7.1	-6	-5.6	-5.1
Apomorphine	-10.4	-7.9	-8.5	-6.7	-6.8	-7.3	-7	-5.9	-5.4	-5.3
Emetine	-9.9	-8.6	-7.5	-7.5	-7	-6.7	-6.8	-5.7	-5.7	-5.5
EGCG	-9.5	-8.4	-8.5	-7.5	-7.2	-6.9	-6.5	-5.7	-5.4	-4.9
Ginsenoside Re	-8.2	-8.5	-8.9	-7.5	-7.2	-6.8	-6.5	-6.1	-5.6	-5.1
Levonorgestrel	-7.9	-8.7	-7.4	-7.4	-6.9	-6.9	-7.3	-6.5	-6.4	-5
Levonorgestrel	-7.9	-8.7	-7.4	-7.4	-6.9	-6.9	-7.3	-6.5	-6.4	-5
Ellipticine	-10.7	-7.7	-7.8	-6.9	-6.7	-7	-6.9	-6	-5.6	-5
Pyrene	-10.1	-7.4	-7.7	-7.1	-6.7	-7.4	-6.6	-5.7	-5.8	-5.8
Doxorubicin	-9.1	-7.9	-8.4	-7.6	-7.3	-6.7	-7.2	-6.3	-4.6	-5.1
Rotenone	-8.3	-8.1	-8.4	-7.2	-7	-7.4	-6.9	-6	-5.4	-5.4
Estradiol	-9.8	-7.7	-7.8	-7.1	-6.9	-7.2	-6.7	-5.9	-5.8	-5.1
Scriptaid	-10.3	-8.1	-7.7	-6.8	-6.7	-6.9	-6.2	-6.1	-5.7	-5.4
Mebendazole	-9.3	-8.1	-7.8	-6.7	-6.9	-7.2	-6.8	-5.9	-6.1	-5.1
Ursodeoxycholic Acid	-8.5	-8.7	-7.8	-7.5	-7.4	-6.4	-6.5	-5.9	-6	-5.2
Pimavanserin	-9.7	-8.4	-7.4	-6.8	-7.1	-6.9	-6.1	-6.4	-6	-4.9
Opicapone	-9.1	-8.5	-8	-7.2	-7.1	-7.5	-6.4	-5.6	-5.1	-5
Fisetin	-9.5	-7.6	-8	-7.1	-6.6	-7.2	-6.4	-6.3	-5.4	-5.1
Puromycin	-9.1	-8.1	-8.1	-7.4	-6.9	-7.1	-6.4	-5.9	-4.8	-5.4
Lutein	-7.6	-9.6	-7	-7.8	-7.2	-7.3	-6.2	-5.7	-5.7	-5
Mirtazapine	-9.3	-7.7	-7.5	-7	-7.1	-6.9	-7.1	-6.1	-5.4	-4.9
LY 294002	-9.8	-7.8	-7.6	-6.8	-7.3	-6.6	-6.8	-6	-5.4	-4.9
Scutellarein	-9.3	-7.6	-7.9	-6.8	-7	-7.4	-6.6	-5.7	-5	-5.1

Table S7. The 3-dimension view of strong binding interactions between targets and drugs

Potential Targets and Ligands	Binding Affinity (kCal/mol)	The 3d View and Interactions of Complex	Target-Ligand Interaction highlighting targeted residues

<table>
<tr><td>(a)

MAPK3
And
Nilotinib</td><td>-12.1</td><td>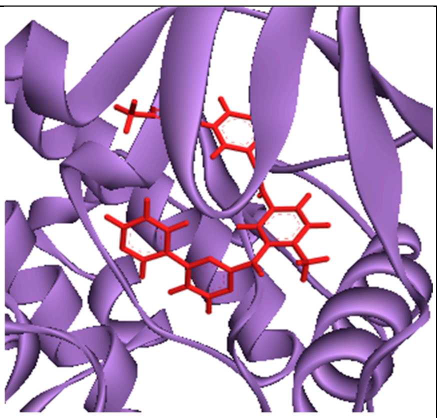</td><td>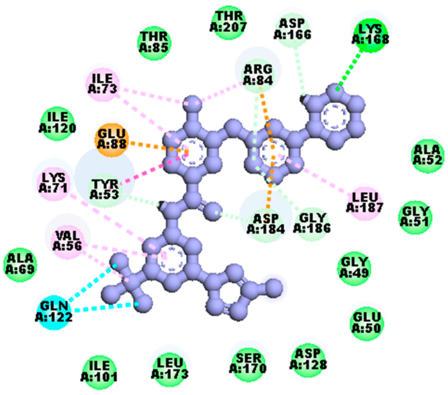</td></tr>
<tr><td>(b)

PTEN
and
Withaferin-A</td><td>-9.7</td><td>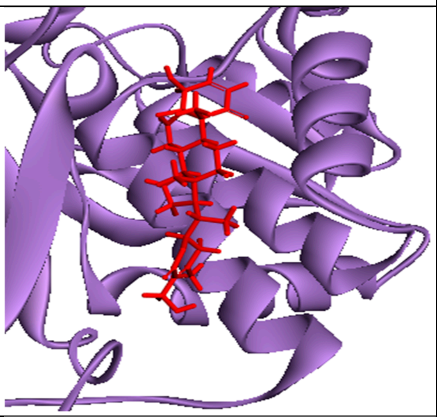</td><td>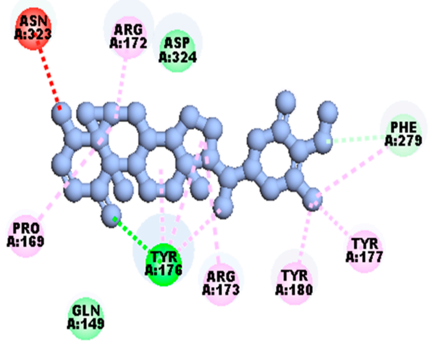</td></tr>
<tr><td>(c)

GAPDH
and
Bromocriptine</td><td>-9.4</td><td>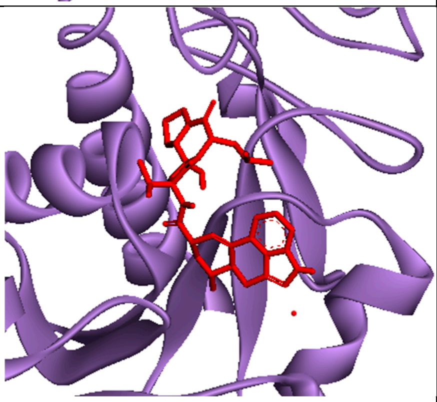</td><td>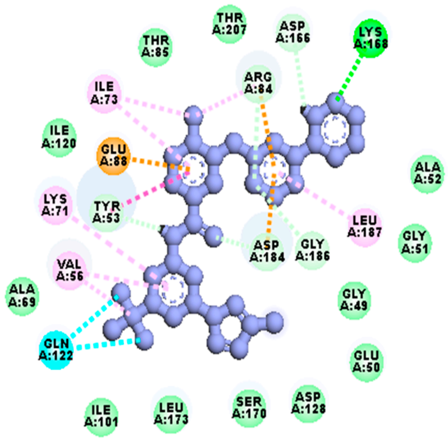</td></tr>
</table>

References

1. Morgan, J.C. and K.D. Sethi, *Emerging drugs for Parkinson's disease.* Expert Opinion on Emerging Drugs, 2006. **11**(3): p. 403-417.
2. Whitney, C.M., *Medications for Parkinson's disease.* The Neurologist, 2007. **13**(6): p. 387-388.

3. Schwab, R.S., et al., *Amantadine in the treatment of Parkinson's disease.* Jama, 1969. **208**(7): p. 1168-70.
4. Rondón-Villarreal, P. and W.O.C. López, *Identification of potential natural neuroprotective molecules for Parkinson's disease by using chemoinformatics and molecular docking.* J Mol Graph Model, 2020. **97**: p. 107547.
5. Agúndez, J.A., et al., *Anti-Parkinson's disease drugs and pharmacogenetic considerations.* Expert Opin Drug Metab Toxicol, 2013. **9**(7): p. 859-74.
6. Hubble, J.P., *Novel drugs for Parkinson's disease.* Medical Clinics of North America, 1999. **83**(2): p. 525-536.
7. McOmish, C., et al., *Muscarinic receptor binding changes in postmortem Parkinson's disease.* J Neural Transm (Vienna), 2017. **124**(2): p. 227-236.
8. Yoo, M., et al., *DSigDB: drug signatures database for gene set analysis.* Bioinformatics, 2015. **31**(18): p. 3069-3071.
9. Athauda, D. and T. Foltynie, *Drug repurposing in Parkinson’s disease.* CNS drugs, 2018. **32**(8): p. 747-761.
10. Griffith, M., et al., *DGIdb: mining the druggable genome.* Nature methods, 2013. **10**(12): p. 1209-1210.
